# Downregulation of N-terminal acetylation triggers ABA-mediated drought responses in *Arabidopsis*

**DOI:** 10.1038/ncomms8640

**Published:** 2015-07-17

**Authors:** Eric Linster, Iwona Stephan, Willy V. Bienvenut, Jodi Maple-Grødem, Line M. Myklebust, Monika Huber, Michael Reichelt, Carsten Sticht, Simon Geir Møller, Thierry Meinnel, Thomas Arnesen, Carmela Giglione, Rüdiger Hell, Markus Wirtz

**Affiliations:** 1 Centre for Organismal Studies, University of Heidelberg, Heidelberg 69120, Germany; 2 Hartmut Hoffmann-Berling International Graduate School, University of Heidelberg, Heidelberg 69120, Germany; 3 Institute of Integrative Biology of the Cell (I2BC), CEA, CNRS, Université Paris-Sud, Bâtiment 21, 1 avenue de la Terrasse, Gif-sur-Yvette F-91198, France; 4 Center for Organelle Research, University of Stavanger, Stavanger N-4036, Norway; 5 Department of Molecular Biology, University of Bergen, Bergen N-5020, Norway; 6 Max Planck institute for Chemical Ecology, Jena 07745, Germany; 7 Center for Medical Research, Mannheim 68167, Germany; 8 Department of Biological Sciences, St John's University, New York, New York 11439, USA; 9 Norwegian Centre for Movement Disorders, Stavanger University Hospital, Stavanger 4068, Norway; 10 Department of Surgery, Haukeland University Hospital, Bergen N-5021, Norway

## Abstract

N-terminal acetylation (NTA) catalysed by N-terminal acetyltransferases (Nats) is among the most common protein modifications in eukaryotes, but its significance is still enigmatic. Here we characterize the plant NatA complex and reveal evolutionary conservation of NatA biochemical properties in higher eukaryotes and uncover specific and essential functions of NatA for development, biosynthetic pathways and stress responses in plants. We show that NTA decreases significantly after drought stress, and NatA abundance is rapidly downregulated by the phytohormone abscisic acid. Accordingly, transgenic downregulation of NatA induces the drought stress response and results in strikingly drought resistant plants. Thus, we propose that NTA by the NatA complex acts as a cellular surveillance mechanism during stress and that imprinting of the proteome by NatA is an important switch for the control of metabolism, development and cellular stress responses downstream of abscisic acid.

## Abstract (web summary)

N-terminal acetylation is a common protein modification in eukaryotes. Here the authors show that in *Arabidopsis*, N-terminal acetylation is decreased by drought stress, that abundance of an N-terminal acetyltransferase is reduced by abscisic acid and that constitutive downregulation can confer drought resistance.

## Graphical abstract

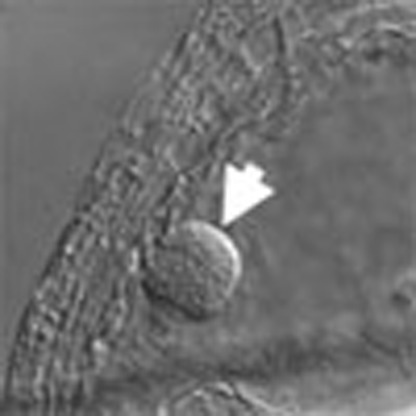

N-terminal acetylation (NTA) is a common modification of eukaryotic proteins occurring on more than 50 and 80% of yeast and human cytosolic proteins, respectively^1^. NTA is catalysed by ribosome-associated N-terminal acetyltransferases (Nats), when 25–50 amino acids of the nascent chain protrude from the ribosome^2^. Three major Nat complexes, NatA, NatB and NatC, accept acetyl-CoA as the donor for the transferred acetate moiety, are present in yeast and humans and are thought to be responsible for the majority of NTA events^1^,^3^. NTA may occur on the initiator Met (iMet) or on the first residue after iMet cleavage by methionine aminopeptidases^4^. NatA potentially acetylates Ser-, Ala-, Thr-, Val-, Gly-, and Cys- N termini after iMet-cleavage^1^, thus, NatA is the predominant Nat with respect to the number of substrates in yeast and human^1^. NatB and NatC potentially acetylate Met- N termini when the second residue is either acidic or hydrophobic, respectively^5^. In yeast and humans, NatA is composed of the catalytic subunit NAA10 (Ard1) and NAA15 (Nat1), anchoring the NatA complex to the ribosome^2^,^5^.

Besides a few examples for specific functions in a handful of proteins (reviewed in ref. 5), the main function of NTA is supposed to be the targeting of misfolded proteins or excessive subunits of protein complexes for ubiquitin-dependent proteasomal degradation^6^,^7^. In these proteins, the acetylated N terminus acts as a preformed degradation signal, called the Ac/N degron, which must be unshielded before it can be recognized by the ubiquitin ligase, DOA10 (ref. 6). Several endogenous yeast proteins have been shown to bear an Ac/N degron in wild-type yeast, but are degraded in *natC* mutants in an Arg/N end rule pathway-based manner^8^.

Although deacetylases of protein N termini have not been identified so far, 9% of all experimentally identified N termini are only partially acetylated in human cells^1^. The partial acetylation might be explained by constitutive poor identification of these substrate N termini by the respective Nats or by differential regulation of Nat activity. Indeed, the interaction of NAA10 with NAA15 in the NatA complex can modulate the active site of Naa10 for substrate-specific NTA^9^, but this interaction is mandatory for ribosome attachment. Thus, no regulatory mechanisms have been evidenced for ribosome-associated Nats so far. The only known factor that can limit co-translational NatA activity is supply with cytosolic acetyl-CoA^10^.

Total loss of NatA, NatB or NatC mutants of humans are not known. However, a point mutation in human NAA10 causes lethality in male infants due to NTA deficiency^11^, which demonstrates the essentiality of NatA for humans. Mutations in the gene encoding the presumed orthologue of human NAA10 have been shown to result in embryonal defects in *Drosophila melanogaster*^12^ and *Arabidopsis thaliana*^13^. In contrast, all studied yeast *nat* mutants are viable; thus, NatA, NatB and NatC are not essential in this unicellular organism. Yeast mutants of the major Nats display a number of different phenotypes suggesting that these Nats, and thus probably NTA in general is implicated in numerous cellular processes reviewed in ref. 5. The discrepancy between phenotypes of NatA-depleted human cells and yeast *natA* provides evidence for relevant differences in the biological function of NTA by the NatA complex in yeast and higher metazoans.

In spite of the fact that NTA is a very common modification in *Arabidopsis*^14^, the significance of NTA in phototrophic eukaryotes is almost unaddressed. *Arabidopsis* T-DNA insertion mutants for genes encoding putative NatB subunits^15^ or the catalytic subunit of NatC (NAA30, Mak3p) were viable but displayed a pleiotropic growth retarded phenotype^16^. However, neither enzymatic activities of putative plant Nat complexes have been demonstrated *in vitro*, nor an *in vivo* substrate of a distinct plant Nat complex has been experimentally identified. Thus, the current knowledge on the plant NTA machinery is based on homology to characterized yeast and human Nat complexes.

Drought is the main reason for crop failure in world agriculture and provokes annually up to 50% of yield losses caused by environmental factors. Consequently, drought stress tolerance of crops was a significant trigger for total yield in the last decades and its significance for yield is supposed to even increase in the future as a result of global warming^17^. The drought stress response of plants is mainly controlled by the phytohormone abscisic acid (ABA), which regulates the transcription of drought-stress-related genes, closure of stomata and root branching^18^,^19^. Ubiquitin-dependent proteasomal degradation is of particular importance for successful drought stress response in plants, since it regulates turnover of many ABA-controlled transcription factors^20^ and removes dehydration-induced malfolded proteins. The latter avoids aggregation of the damaged proteins and ensures proteostasis, which is under control of sophisticated cellular surveillance mechanisms in eukaryotes reviewed in ref. 21. These cellular surveillance mechanisms not only control proteolysis but also translation and correct folding of proteins at the ribosome. Thus, the ribosome has been proposed recently as a central hub for protein quality control in eukaryotes^21^. Here we ask the question if co-translational NTA of proteins by the ribosome-attached NatA complex contributes to cellular surveillance during stresses in higher plants and address the biological significance of NatA activity for regulation of metabolism and development.

We demonstrate that NAA10 and NAA15 are mandatory for NatA activity and target nearly 50% of soluble proteins in leaves. Thus, composition and substrate specificity of the NatA complex is evolutionary conserved within higher eukaryotes. Plant NatA activity is essential for proper embryogenesis and controls drought stress-induced developmental plasticity of the root system. Surprisingly, the turnover of the NatA complex is under the control of the stress-dedicated phytohormone ABA. Decrease of NatA is found to be sufficient for the induction of the canonical drought stress response and downregulates many biosynthetic pathways. Thus, we propose that imprinting of the cytosolic proteome by NatA is a master switch for the control of metabolism, development and cellular stress responses and integrates stress signals by perceiving the hormone ABA.

## Results

### Biochemical characterization of the *Arabidopsis* NatA complex

A search for orthologous proteins of the NatA complex in *Arabidopsis* with the TAIR BLASTP 2.2.17 algorithm (www.arabidopsis.org) revealed two candidates, At5g13780 (AtNAA10; AY091419) and At1g80410 (AtNAA15; AY087018), which show 67 and 41% identity with the human Naa10 and Naa15 proteins, respectively. Transcription of *AtNAA10* and *AtNAA15* was verified by quantitative reverse transcription–PCR (qRT–PCR) in roots, leaves, stem and flowers of *Arabidopsis* (Supplementary Fig. 1a). Purified recombinant AtNAA10 protein (MBP-His-AtNAA10) acetylated specifically an oligopeptide with a known NatA-type substrate sequence (STPD (ref. 1)) (Supplementary Fig. 1b). Formation of NatA complex was independently demonstrated by co-immunoprecipitation of full-length AtNAA10 and AtNAA15 in fusion with V5 tag and Xpress tag (Supplementary Fig. 1c), a bimolecular fluorescence complementation (BiFC) assay, which also revealed cytoplasmic localization of both proteins (Supplementary Fig. 1d) and a yeast two-hybrid approach (Supplementary Fig. 1e). Separation of soluble *Arabidopsis* leaf proteins by differential ultracentrifugation revealed co-fractionation of AtNAA15 with the ribosomal protein S14, strongly suggesting the association of the auxiliary subunit AtNAA15 with ribosomes (Supplementary Fig. 1f). Thus, the biochemical determinants and the biological function of NatA subunits are conserved between the metazoan and the plant NatA complex.

### The NatA complex is essential for *Arabidopsis* embryogenesis

**Figure 1:**
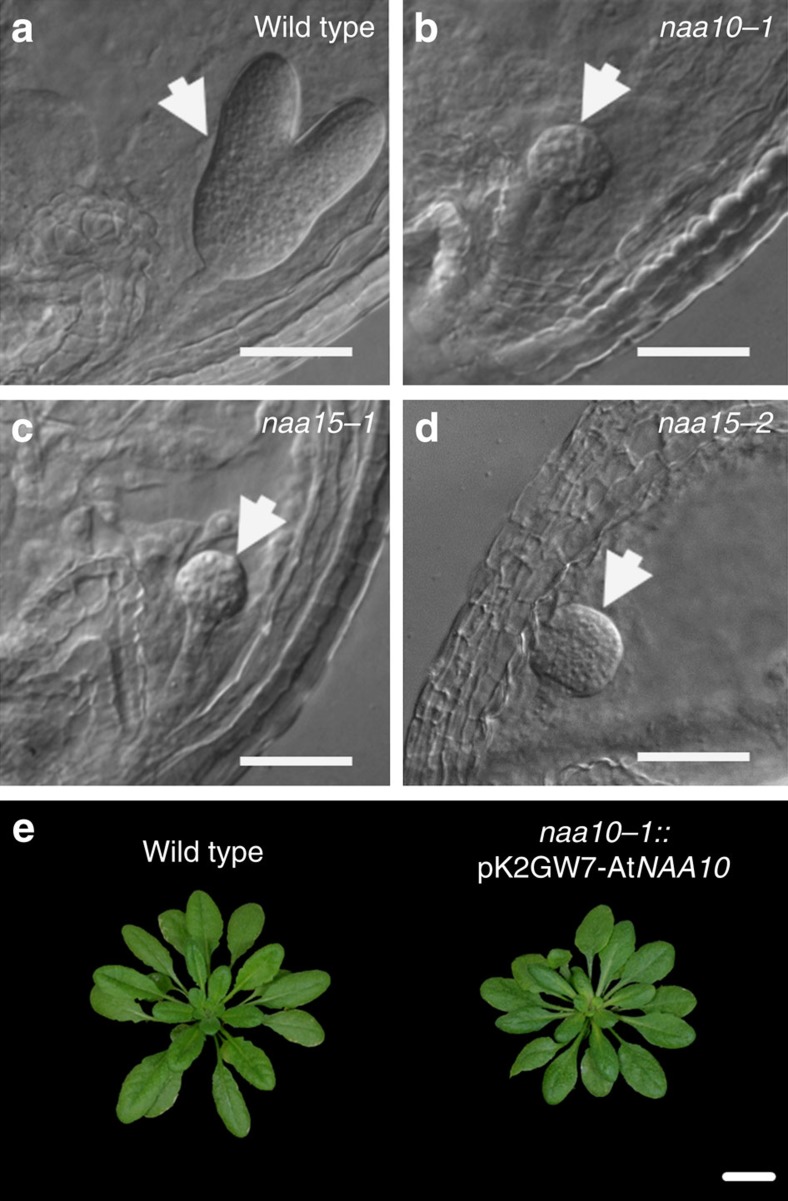
Loss of the NatA complex causes embryo lethality. (**a–d**) Differential interference contrast microscopy (DICM) of developing embryos (arrows) in wild-type (**a**), *naa10-1* (**b**), *naa15-1* (**c**) and *naa15-2* (**d**) plants 14 days after flowering. Plants were grown and seed pods embedded for DICM as described in Supplementary Methods. (**e**) Phenotype of 8-week-old wild-type and homozygous *naa10-1* plants that ectopically express AtNAA10 (pK2GW7-AtNAA10). Only one representative pK2GW7-AtNAA10 line out of five successfully complemented transgenic homozygous naa10-1 lines is shown. Scale bar, 0.1 mm (**a**–**d**) or 2 cm (**e**).

The *in vivo* function of NTA by the NatA complex in *Arabidopsis* was investigated by T-DNA insertion alleles for *AtNAA10* (*naa10-1*) and *AtNAA15* (*naa15-1*, *naa15-2*). Using differential interference contrast microscopy, we found that on heterozygous *naa10-1* plants 20% of analysed embryos (*n*=120) arrested at the dermatogen to early globular stage, demonstrating that loss of *NAA10* activity causes embryo lethality (Fig. 1a,b). Homozygous *naa15-1* (25%, *n*=236) and *naa15-2* (22%, *n*=60) also arrested at the same embryonic stage (Fig. 1c,d). Genetic complementation of homozygous *naa10-1* plants with AtNAA10 under control of the CaMV35S promoter rescued the embryo lethal *naa10-1* phenotype and results in wild-type-like growth of complemented *naa10-1* plants, confirming the lack of NAA10 as single cause of the observed phenotype (Fig. 1e, Supplementary Fig. 2).

### Downregulation of NatA complex results in retarded growth

**Figure 2:**
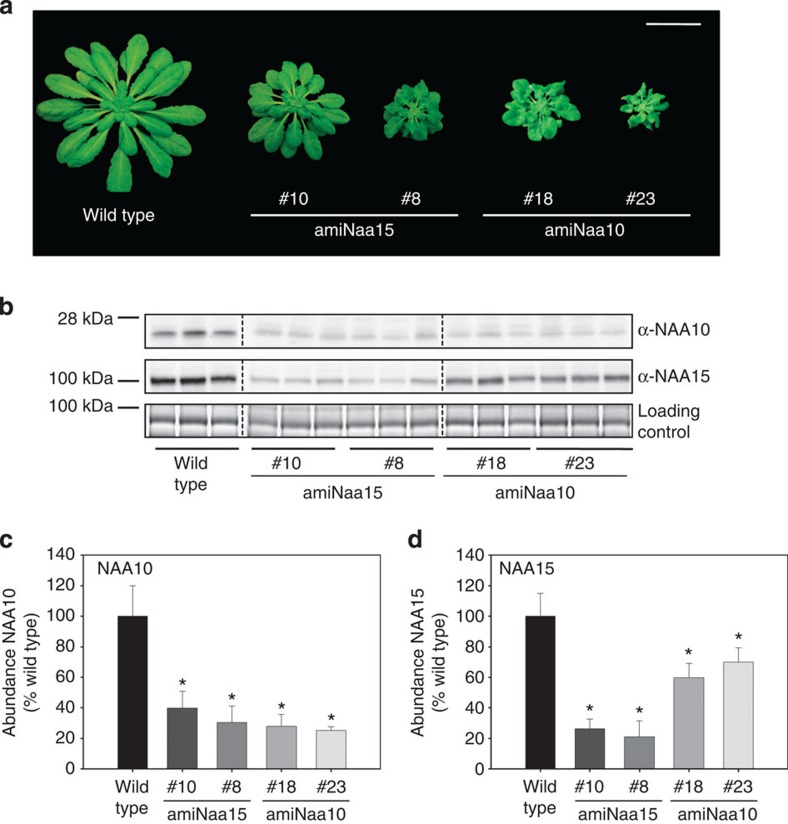
Depletion of NAA10 and NAA15 results in the same retarded vegetative growth phenotype. (**a**) Growth phenotype of amiNaa15 lines 10 and 8 and amiNaa10 lines 18 and 23 grown for 7 weeks on soil under short-day conditions. (Scale bar, 4 cm) (**b**) Immunological detection of NAA10 or NAA15 with specific antisera in leaves of NatA-depleted plants. Coomassie blue-stained protein served as loading control. Dashed lines indicate rearrangement of immunological signals. The original blot is shown in the Supplementary Fig. 7. (**c**,**d**) Quantification of NAA10 (**c**) and NAA15 (**d**) signals shown in **b**. Data are represented as mean±s.d. Asterisks indicate significant differences (*P*<0.05, *N*=3, Student's *t*-test).

To overcome the embryo lethal phenotype of the NatA loss-of-function mutants, artificial microRNAi sequences directed against *NAA10* or *NAA15* were expressed under the control of the CaMV35S promoter in stably transformed wild-type *Arabidopsis* (ecotype Col-0). A series of independent transgenic lines harbouring pBin-amiNAA10 (amiNaa10) and pBin-amiNAA15 (amiNaa15) were identified and displayed a significant reduction of growth. Transcription of the artificial microRNA (21 bp) against *NAA10* was verified by RNA–DNA hybridization and qRT–PCR in selected amiNaa10 plants and demonstrated a strong correlation between steady-state level of the NAA10 microRNA and growth retardation (Supplementary Fig. 3). We selected two independent lines for amiNaa10 and amiNaa15 that have intermediate to strong growth phenotypes (Fig. 2a) for further analyses. Downregulation of the *NAA15* transcript in the amiNaa15 lines 8 and 10 and the *NAA10* transcript in the amiNaa10 lines 18 and 23 resulted in significant decrease of Naa10 and Naa15 protein level, respectively (Fig. 2b,c). Interestingly, downregulation of the catalytically active or the auxiliary subunit of NatA by the artificial microRNA caused also decreased the abundance of the second subunit of the NatA complex (Fig. 2b,c). Analysis of individual amiNaa15 plants revealed significant correlation between decrease of NAA15 abundance and growth retardation (Supplementary Fig. 3, *r*^2^=0.99). Despite of the growth retardation, all isolated transgenic lines were fertile.

### Depletion of NatA increases free N termini in plants

**Figure 3:**
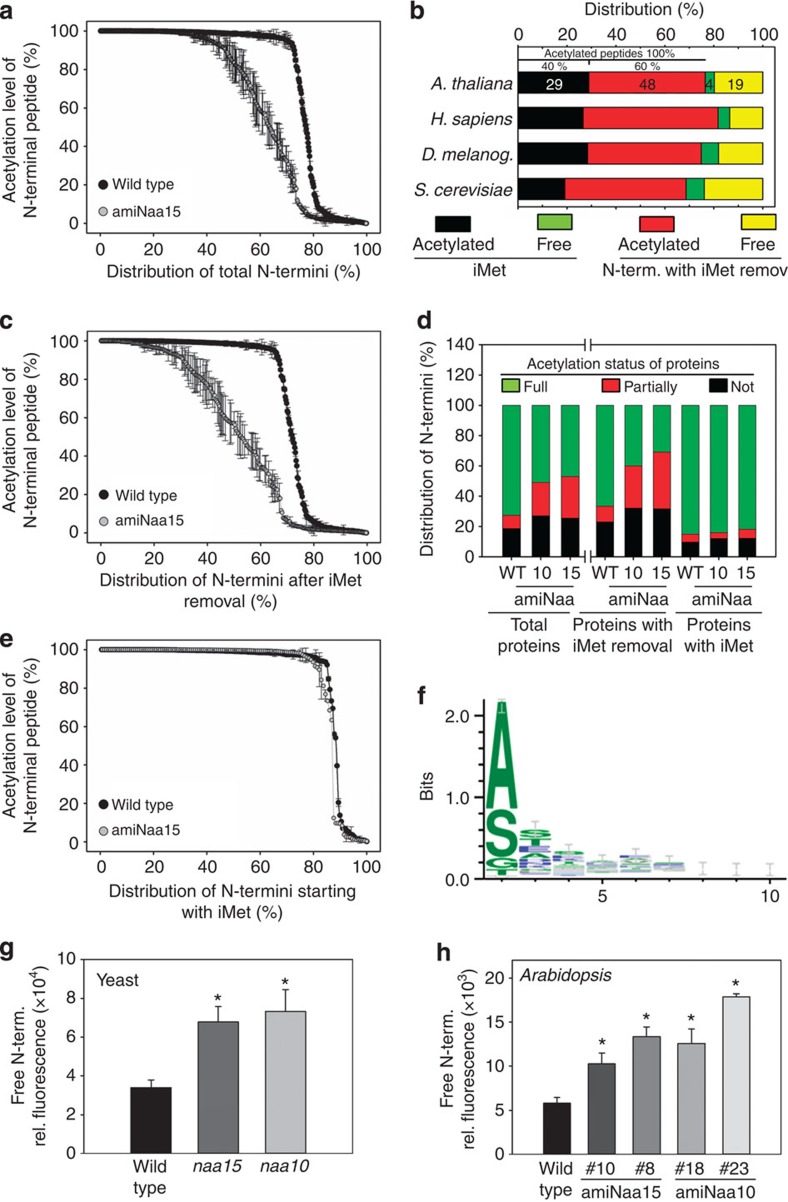
The plant NatA complex targets N termini after iMet removal. (**a**) Acetylation level of identified N-terminal peptides in leaves of 7-week-old wild-type (black) and amiNaa15 (grey) plants. (**b**) Amount of acetylated (black, red) and not acetylated N-terminal peptides (green, yellow) that were subject of iMet excision (red, yellow) or start with iMet (black, green) in *Arabidopsis* and other eukaryotes. (**c**,**e**) Acetylation level of N-terminal peptides from proteins that are subject to removal of iMet (**c**) or that are starting with iMet (**e**) in wild-type and amiNaa15 plants (subset of **a**). (**d**) Comparison of acetylation status for all proteins, proteins starting with iMet and proteins that were subject of iMet removal from wild-type and NatA-depleted plants. Proteins are designated as fully acetylated (green), when more than 90% of N-terminal peptides were found to be acetylated. Proteins of which 10% or less of N-terminal peptides were found acetylated are defined not acetylated (black). The remaining subset of proteins (20–80% acetylated N-terminal peptides) was defined partly acetylated. Soluble proteins were extracted from leaves of wild type, amiNaa10 line 23 and amiNaa15 line 8. After digestion of proteins, N-terminal peptides were enriched by SCX-approach for MS/MS analysis (*N*=4). (**f**) Weblogo of the experimentally characterized protein N termini that were subjected to iMet removal and highlighting differential NTA yield between WT versus amiNAA15 plant samples. (**g**) Quantification of free N termini in soluble protein extracts of the wild-type yeast and loss-of-NatA function mutants (*naa15* and *naa10*) using NBD-Cl according to Supplementary Methods. (**h**) Quantification of free N termini in soluble protein fraction of 7-week-old-wild-type and NatA-depleted plants using the same method. Data are represented as mean±s.d. Asterisks indicate significant differences (*P*<0.05, *N*=3–4, Student's *t*-test).

Characterization of more than 4,500 N-terminal peptides from 1,161 distinct non-redundant proteins by MS/MS analysis suggested that the phenotype of NatA-depleted mutants is caused by significant decrease of protein NTA (Fig. 3a, Supplementary Fig. 4a). Seventy-seven per cent of the characterized N termini in the leaves of wild-type *Arabidopsis* are modified by NTA (Fig. 3b, black+red). The minor fraction (40%) of these acetylated peptides is acetylated at the iMet (29% of all characterized N termini, Fig. 3b, black). The majority of experimentally identified acetylated N termini (60%) in *Arabidopsis* is acetylated after removal of the iMet and display at the second position A>S>G>T (Fig. 3f). This subset of protein N termini is potentially targeted by NatA and represents about 47% of the identified *Arabidopsis* leaf proteome (Fig. 3b, red). The specific decrease in acetylation of this subset of N termini (Fig. 3c) triggered the decrease of total acetylation level in amiNaa10 (Supplementary Fig. 4a) and amiNaa15 lines (Fig. 3d) and is therefore considered as NatA substrates (148 N termini, Supplementary Data 1). Acetylation level of N termini identified with an iMet was almost not affected (only five N termini displayed decreased acetylation level, Supplementary Data 1) in the mutants suggesting the acetylation of these substrates is due to the combined activity of NatB, NatC and NatF (Fig. 3d,e). NatA substrates (78%) are considered fully acetylated (acetylation yield >90%), while 12% of identified NatA-type N termini were found not acetylated (acetylation yield <10%) in the wild type. Interestingly, 10% of the NatA substrates are partially acetylated in wild-type plants. Downregulation of NAA10 and NAA15 increased the number of not and partially acetylated NatA substrates to a similar extent in both mutants and resulted in a decrease of fully acetylated NatA substrate from 78% in the wild type to 30% (amiNaa10 line 23) or 34% (amiNaa15 line 8) in the mutants. The here-applied MS/MS approach for quantification of protein N termini did not reveal lowered acetylation of premature N termini of nuclear-encoded organellar proteins (Supplementary Data 1), since these N termini are unstable and thus hardly detected.

The distribution between acetylated amino-acid residues at position 2 of experimentally confirmed acetylated NatA substrates (A>S>G,T) is most similar between *Arabidopsis* and humans. Both distributions differ significantly from the distribution within NatA substrates in yeast (S>A>G,T, Supplementary Fig. 4b). NatA substrates of *D. melanogaster* display an intermediate kind of distribution for preference of the second amino-acid residue. Also the overall distribution of the acetylome substrates specificity show higher similarity between higher eukaryotes (*D. melanogaster*^22^, *Arabidopsis* and human^1^) when compared with lower eukaryote yeast^1^ (Fig. 3b).

To allow for direct quantification of free N termini in NatA-depleted plants, we established a fast and sensitive assay for the detection of free N^α^-termini of proteins that is based on the specific labelling of N^α^-terminal group of polypeptides with the fluorescent dye, 4-chloro-7-nitrobenzofurazan (NBD-Cl)^23^. The suitability of the optimized assay for N^α^-terminal groups in complex mixtures of non-denatured soluble proteins was demonstrated by detection of an about twofold increase in free N^α^ termini of native soluble proteins of the yeast NatA loss-of-function mutants *Scnaa15* (*nat1*) and *Scnaa10* (*ard1*; Fig. 3g). This is in agreement with earlier studies demonstrating that NatA approximately acetylates half the yeast proteome^1^. All analysed transgenic plants with decreased NAA15 or NAA10 subunits displayed a significant increase of free N termini that was in the same order compared with the yeast NatA loss-of-function mutant (Fig. 3h). Furthermore, the increase in detected free N termini correlated with the degree of NatA subunit depletion and the retardation of growth (Fig. 2).

### NatA-depleted plants are highly drought tolerant

**Figure 4:**
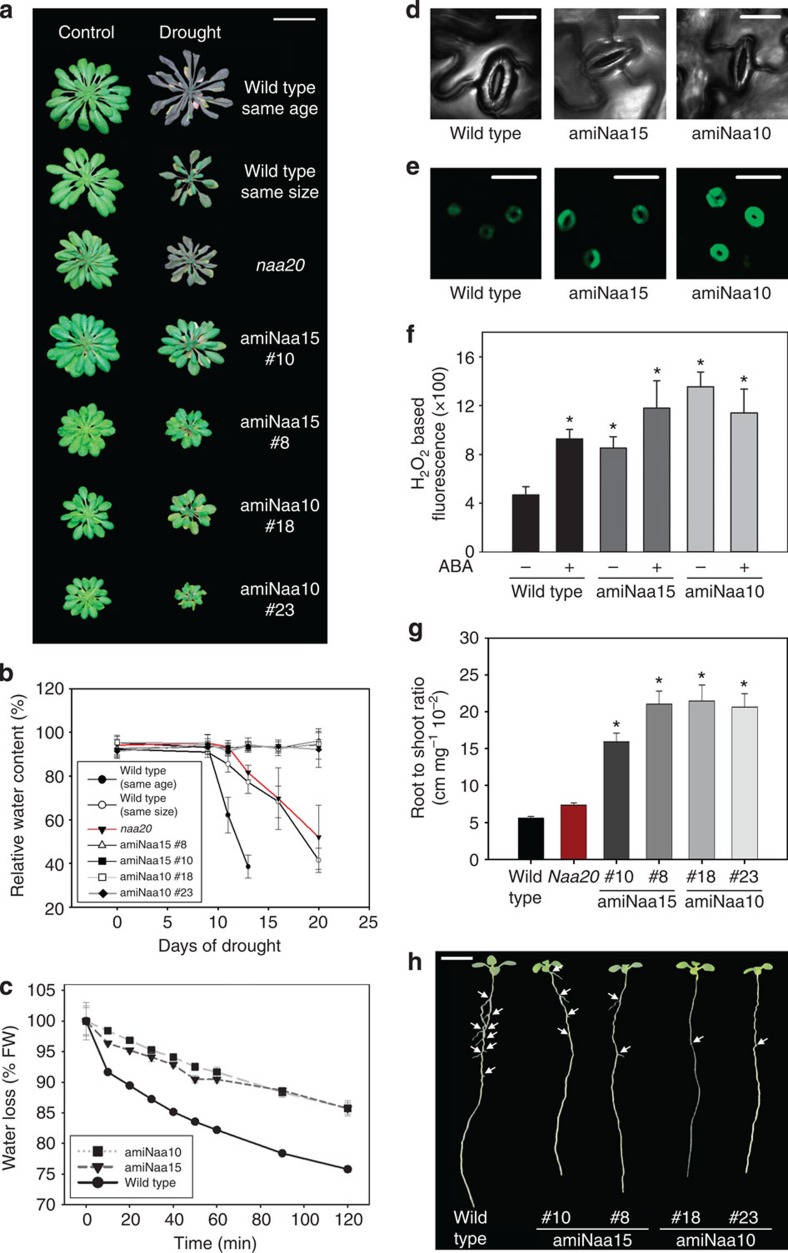
NatA-depleted *Arabidopsis* plants display drought tolerance. Wild-type (same age) and NatA-depleted plants were grown for 42 days on soil under short-day conditions and subsequently challenged with drought for 20 days. Twety-eight-day-old wild-type plants (same size) served as an additional control to test the impact of developmental stage on the tolerance against drought. (**a**) Growth phenotype of wild-type and NatA-depleted plants grown under control conditions or drought stress for 20 days (Scale bar, 4 cm). (**b**) RWC in leaves of wild-type, amiNaa10 and amiNaa15 plants during application of drought stress. Data are represented as mean±s.d. (*N*=4). (**c**) Transpiration of leaves from 6-week-old wild-type and NatA-depleted plants. Data are represented as mean±s.d. (*N*=3). (**d**) Top view on single stomata from wild-type and NatA-depleted plants (Scale bar, 20 μm). (**e**) Fluorescent staining of hydrogen peroxide in guard cells of wild-type and NatA-depleted plants (Scale bar, 40 μm). (**f**) Quantification of hydrogen peroxide-dependent fluorescence by confocal laser scanning microscopy in guard cells of wild type and NatA depleted in absence or presence of 50 μM ABA. Wild type and NatA depleted were grown on soil under short-day condition with regular water supply for 6 weeks before analysis. Data are represented as mean±s.e. Asterisks indicate significant differences to wild type. (*P*<0.05, *N*=10–80, Student's *t*-test) (**g**) Root-to-shoot ratio of wild-type, *naa20* and NatA-depleted plants grown hydroponically on ½ Hoagland medium for 6 weeks under short-day conditions. Asterisks indicate significant differences to wild type determined by Student's *t*-test. Data are represented as mean±s.d. (*P*<0.05, *N*=30–35), Student's *t*-test). (**h**) Root phenotype of 2-week-old wild-type and NatA-depleted plants grown under short-day conditions on solid ½ MS-medium (Scale bar, 0.5 cm). Arrows indicate lateral roots.

The hypothesis of NTA-dependent quality control of proteins^7^ prompted us to test if the NatA complex is involved in response to stresses. The NatA-depleted mutants were compared with NatB T-DNA insertion mutant (*naa20*) and wild type of the same age and the same developmental stage and in a time series for its drought resistance. Noteworthy, the *naa20* mutant displays a similar decrease in growth rate as NatA-depleted plants^15^. NatA-depleted plants were significantly more resistant to drought than both wild-type controls and *naa20*, demonstrating that the slower growth of NatA-depleted mutants is not the reason for the higher drought resistance compared with wild type (Fig. 4a). The relative water content (RWC) of leaves decreased in the two wild-type controls as result of prolonged drought (day 10 to 20, Fig. 4b). After 20 days of drought, wild-type plants died and could not be recovered by resupply of water (Supplementary Fig. 5a,b). In contrast, all tested amiNaa10 and amiNaa15 lines were viable after 20 days of drought, did not show any visible drought-related phenotype and completed life cycle after resupply of water (Supplementary Fig. 5b). Astonishingly, the NatA-depleted plants retained almost 95% RWC even after 20 days of drought, which explains the drought-tolerant phenotype (Fig. 4b) and the soil water content was higher in pots that contained NatA-depleted plants (Supplementary Fig. 5c).

### Depletion of NatA activity causes closure of stomata

The capability of NatA-depleted plants to retain almost all water even after 20 days of drought prompted us to test the transpiration rate of truncated leaves in a short-term experiment. Detached leaves of amiNaa10 and amiNaa15 lines lost significantly less water over time than detached wild-type leaves (Fig. 4c). Transpiration of leaves is controlled by abundance and aperture of stomata in higher plants. The density of stomata was unaffected in leaves of NatA-depleted plants (Supplementary Fig. 5d), but the stomata aperture in the NatA-depleted mutants was significantly decreased by ∼2 μm, which represents the almost fully closed state of stomata (Fig. 4d, Supplementary Fig. 5e). In search for a functional explanation for the closure of stomata in NatA-depleted plants, we quantified hydrogen peroxide (H_2_O_2_) in guard cells of stomata (Fig. 4e). H_2_O_2_ is a canonical trigger for stomata closure and its production is known to be regulated in plants by the hormone ABA. Guard cells of NatA-depleted plants display two to three times higher steady-state levels of reactive oxygen species (ROS) than the wild-type guard cells in the absence of exogenous applied ABA (Fig. 4e,f). Application of ABA to wild-type leaves increased ROS in guard cells to the same levels as in guard cells of non-treated NatA-depleted plants. The concentration of ROS in guard cells of NatA-depleted plants was not increased further by ABA treatment (Fig. 4f).

### NatA-depleted plants display drought-adapted root morphology

Since drought tolerance is often mediated by improved water uptake by the root system, we analysed the root morphology of the wild type, the *naa20* mutant and the NatA-depleted mutants. NatA depleted showed a significant increase of total root length to shoot fresh weight ratio when compared with wild type and the *naa20* mutant (Fig. 4g). This increase is driven by growth of the primary root in the NatA-depleted plants. The formation of lateral roots is lowered in the NatA-depleted mutants, which results in a significant decrease of lateral root density under non-stressed conditions when compared with wild type (Fig. 4h, Supplementary Fig. 6a). The increase of primary root length and the inhibition of lateral root formation are both known adaptations of the wild type towards drought and are triggered by the phytohormones IAA and ABA^18^ via the transcription factors MYB77 and ARF7 (ref. 24). Transcript steady-state levels of both transcription factors are significantly downregulated in amiNaa10 and amiNaa15 lines (Supplementary Fig. 6b), suggesting that these transcription factors contribute to the observed constitutive drought stress adaptation of root morphology in NatA-depleted plants under non-stressed conditions.

### NatA depletion induces ABA responses

**Figure 5:**
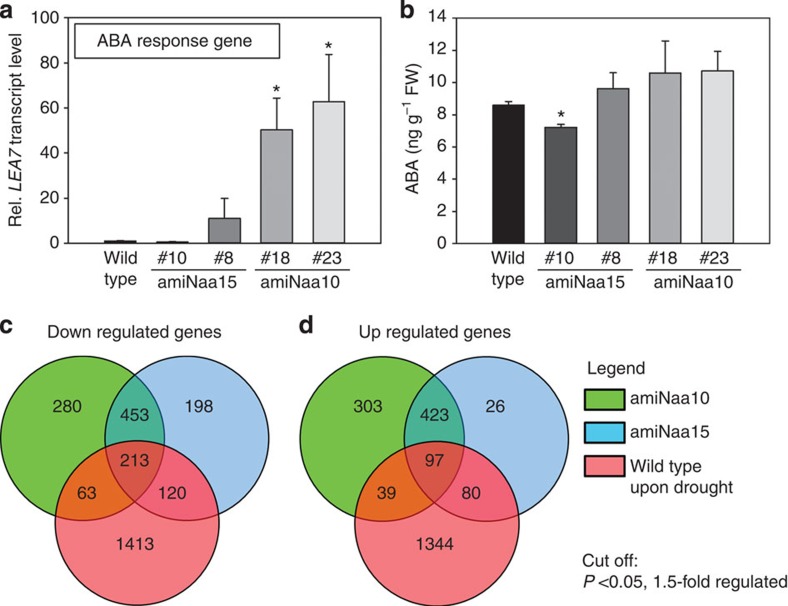
Transcriptional response to NatA depletion. ABA steady-state levels and transcription of ABA-related genes were determined in leaves of 6-week-old wild type and NatA depleted grown on soil with regular water supply. (**a**) Transcript steady-state level of the ABA-inducible marker gene, *LEA7*. (*N*=3) (**b**) Steady-state levels of ABA. (*N*=5) (**a**,**b**) Data are represented as mean±s.e. Asterisks indicate significant differences to wild type. (*P*<0.05, Student's *t*-test). (**c**,**d**) Venn diagrams for comparison of downregulated (**c**) and upregulated genes (**d**) in wild-type plants by drought stress (10 days) with genes that are regulated in NatA-depleted plants under control conditions when compared with wild type. Global transcriptional response in leaves of well-watered amiNaa10 (green), amiNaa15 (blue) was compared with non-stressed wild-type plants (control) as determined by hybridization of total mRNA to the Gene 1.0 ST Array (Affymetrix, Germany). (*N*=4).

Lateral root formation and closure of stomata are triggered by ABA in the wild type on drought. We therefore tested the expression of the well-established ABA marker gene, *LEA7*, in leaves of NatA-depleted plants and the wild type. In the amiNaa10 line 23, which shows strongest depletion of NatA, the steady-state transcript level of *LEA7* was ∼60-times higher than the wild type (Fig. 5a). The increase of *LEA7* transcript steady-state level correlated with the degree of NatA depletion and was not significantly changed in amiNaa15 line 10. To test if *LEA7* transcription is triggered by higher ABA levels in NatA-depleted plants, we quantified ABA levels in leaves and roots of NatA-depleted plants by LC-MS/MS. Neither in the leaves (Fig. 5b) nor in the roots (Supplementary Fig. 6c) a significant increase of ABA could be detected in NatA-depleted plants when compared with wild type.

In search for NatA-regulated processes, we determined the global transcriptome in leaves of NatA-depleted mutants and compared it with the response of the wild type on drought stress. Transcriptomes of amiNaa10 line 23 and amiNaa15 line 8 displayed a remarkable degree of co-regulation (73%), showing that the downregulation of the catalytic and the auxiliary subunit cause a similar transcriptional response in leaves of both mutants. Downregulated genes (32%) and 19% of upregulated genes in NatA-depleted plants are part of the drought stress-induced response in wild-type plants (Fig. 5c,d). A gene annotation enrichment analysis revealed significant downregulation of flavonoid and phenylpropanoid biosynthesis pathways in NatA-depleted plants (Table 1), which both use phenylalanine as precursor. Interestingly, sulfate assimilation was downregulated in leaves of both mutants, but nitrate assimilation was unaffected. This strongly indicates that the downregulation of sulfate assimilation is specific and not a simple consequence of slower growth of NatA-depleted plants. The only pathway that was significantly upregulated in NatA-depleted plants was ubiquitin-mediated proteolysis (Table 1).

### Drought stress and ABA cause increase of free N termini

**Figure 6:**
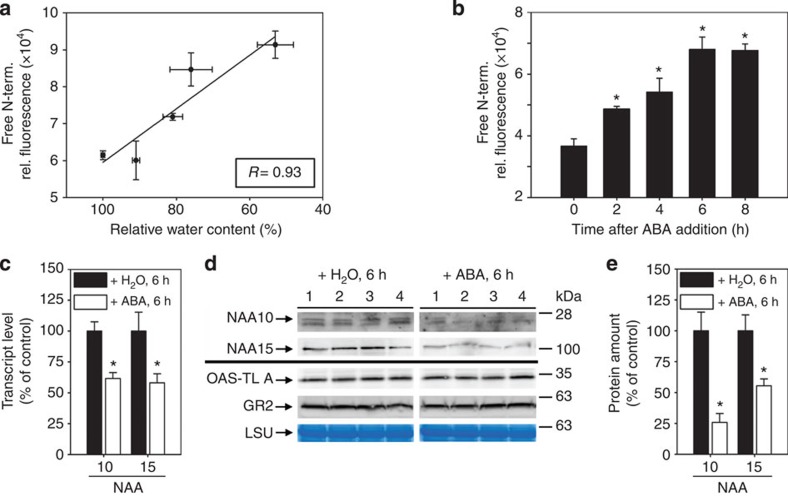
Drought stress and ABA cause significant increase of free N termini. (**a**) Correlation between free N termini of soluble proteins and the RWC determined in leaves from 6-week-old wild-type plants after application of drought (*N*=3–5). (**b**) Quantification of free N termini in soluble proteins extracted from leaf discs of 6-week-old wild-type plants treated with 50 μM ABA for indicated time (*N*=4). (**c**) *NAA10* and *NAA15* transcript steady-state level in the wild-type leaf discs after application of 50 μM ABA for 6 h. (**d**) Immunological detection of NatA subunits and two cytosolic control proteins (OAS-TL A, GR2) in wild-type leaf discs after application of 50 μM ABA for 6 h. Coomassie-stained RuBisC/O large subunit (LSU) served as loading control. (**e**) Quantification of NAA10 and NAA15 signal shown in **d** after normalization to loading control. The amount of NAA10 or NAA15 in wild-type leaf discs treated with water was set to 100% (*N*=4). Data are represented as mean±s.e. Asterisks indicate significant differences to wild type. (*P*<0.05, Student's *t*-test).

Depletion of the NatA complex resulted in higher amount of free N termini on the global scale (Fig. 3) and constitutively induced part of drought stress response in presence of low ABA levels (Figs 4 and 5). We therefore hypothesized that the generation of free N termini might be part of the drought stress response and act downstream of ABA. Quantification of free N termini in soluble protein extracts from leaves of wild type challenged with drought revealed a significant correlation between decrease of RWC and quantity of free N termini (Fig. 6a). Exogenous application of ABA caused a significant increase of free N termini in the soluble protein fraction within 2 h. The amount of free N termini increased linearly for 6 h and reached a plateau after 8 h (Fig. 6b). ABA treatment resulted in significant downregulation of *NAA10* and *NAA15* transcript steady level to ∼60% of wild-type level after 6 h (Fig. 6c). Immunological detection of NAA10 and NAA15 revealed significant and specific decrease of the catalytic (74%) and the auxiliary subunit (45%) of NatA (Fig. 6d,e), while stable cytosolic (OAS-TL A) and plastidic control proteins (GR2) were not affected by short-term ABA treatment (Fig. 6d). Taken together, these data demonstrate that ABA downregulates NatA abundance and causes an increase of free protein N termini within a few hours' time range. Consequently, the amount of free N termini increases on drought stress in wild-type plants.

## Discussion

The here-applied biochemical and reverse genetics approaches demonstrate the identity of At5g13780 as the catalytic subunit NAA10 and At1g80410 as the auxiliary subunit NAA15 of the *Arabidopsis* NatA complex. The first *in vivo* substrate characterization of a Nat complex from a phototrophic eukaryote demonstrates high similarity of the *Arabidopsis* NatA substrate specificity to the substrate specificities of the human and yeast NatA complex^1^,^14^. Thus, NatA acts co-translationally after the excision of iMet by methionine aminopeptidasess in all analysed organisms so far, which is in agreement with complementation of yeast loss-of-NatA mutants by reconstituted human NatA complex. Remarkably, the human catalytic or auxiliary NatA subunits do not complement respective single loss-of-function yeast mutants^1^.

Loss-of-NatA activity in *Arabidopsis* or NAA10 activity in *D. melanogaster*^12^ and humans^11^ is fatal, but causes only slight growth retardation in yeast grown under optimal conditions in full medium^25^. A possible explanation for this discrepancy might be provided by the different distribution of NatA substrates in higher eukaryotes and yeast. While in higher eukaryotes most NatA substrates starts with A>S>T, G, the frequency of these substrates is significantly different in yeast^14^,^26^. The experimentally identified NatA-dependent acetylome of *Arabidopsis* shared highest similarity to the human NatA-dependent acetylome.

A hallmark of higher eukaryotes is multicellularity, which provides the basis for the specification of cells in tissues. This specification is mainly controlled by defined hormone gradients in higher eukaryotes. In the higher eukaryote *Arabidopsis*, NatA activity is regulated by the phytohormone ABA (this work) and the developing NatA-deficient plant embryos arrest exactly in the stage of embryogenesis in which hormone gradients define the apical–basal axis^27^. However, abortion of embryogenesis can be caused by specific deregulation of developmental processes or by defects in primary metabolism. For that reason, arrest of embryogenesis in NatA-deficient *Drosophila* or *Arabidopsis* is indicative, but does not prove the direct involvement of the NatA complex in the regulation of development.

Downregulation of the NatA complex in plants causes not only growth retardation in the vegetative stage but also significantly affects the root-to-shoot ratio and the root morphology (Fig. 4). Both developmental processes are known to be ABA regulated and comprise significant transcriptional adaptation of key regulators that has been also been evidenced in NatA-depleted roots^18^,^24^. In view of the fact that the abiotic factor, drought, impacts the N^α^-acetylome, we hypothesize a mediating role of the NatA complex in the enormous plasticity of plants towards environmental challenges reviewed in ref. 28.

NTA has been designated as co-translational and irreversible imprinting of the proteome, and not considered subject to regulation^29^. NTA imprinting was thought to influence the fate of any given protein constitutively. This dogma is also supported by the lack of proven N-terminal deacetylases in any organism. NTA of proteins was suggested to be important for targeting specific proteins for degradation after malfolding and/or release from multi-subunit complexes by unshielding of a preformed degradation signal^6^,^7^ or to assist proteins in obtaining their proper subcellular localization^30^,^31^. Nonetheless, both suggestions have not taken into account the possible relevance of the partially modified acetylome or even its modulation in response to biotic or abiotic stresses.

Up to now, no reports for active regulation of the NatA acetylome or any other N^α^-acetylome have been reported. The only identified factor that impacts Nat activity was limitation by acetyl-CoA supply, which will affect nonspecifically all Nat complexes and many other biosynthetic routes in the cytosol^10^. Here we show that ∼10% of all NatA substrates are partially acetylated under non-stressed conditions in leaves of *Arabidopsis*. A similar degree of partially acetylated N termini has been also found in humans. Astonishingly, only 16 NatA substrates (6.6% of all identified NatA substrates) were less acetylated in HeLa cells after the downregulation of hNAA10 to 5% of wild-type level^1^. In higher plants, the NatA-dependent acetylome is much more sensitive to regulation of NatA abundance. Depletion of NatA to 20% of the wild-type levels resulted in a significant increase of free and partially acetylated N termini (up to 30% of all identified NatA substrates, 148 proteins) and caused an overall decrease in the NatA acetylation frequency. Such a decrease in acetylation frequency was also found in leaves of water-deprived plants and revealed for the first time a physiological adaptation of the N^α^-acetylome in response to an abiotic stress in higher eukaryotes. In agreement with a regulatory function of protein NTA in plants on stress, a recent study has shown that the ɛ subunit of chloroplast ATP synthase occurs as both acetylated and non-acetylated form and the level of the latter preferentially decreases under drought conditions^32^. Taken together, these results imply a paradigm shift for the biological function of protein NTA.

The first time-resolved quantification of the global acetylation status in a higher eukaryote demonstrates quick adaption of the leaf N^α^-acetylome after application of the stress-related hormone ABA (<6 h). The increase in unacetylated protein N termini was at least partially triggered by fast and significant downregulation of NatA abundance on ABA treatment, which provides a solid explanation for the remarkable correlation between unacetylated N termini and the RWC in leaves. Thus, in higher plants, NTA by NatA is an ABA-regulated dynamic process that adopts the N^α^-acetlyome in response to environmental changes. Such a hormone-regulated adaptation of the N^α^-acetylome on stress has not been reported for any other eukaryote up to now, but is in agreement with the sensitivity of yeast NatA loss-of-function mutants to abiotic stresses like heat shock or nutrient starvation^25^. Remarkably, also NatB and NatC yeast knockout mutants have been shown to be sensitive to specific sets of abiotic stresses^25^.

The molecular reason for the stress-resistant phenotype of NatA-depleted plants is the constitutive induction of major components of the drought stress response. This constitutive induction comprises metabolic adaptation of single cells (increase of H_2_O_2_-induced stomata closure), alteration of global transcriptional response and adjustment of developmental processes (inhibition of lateral root hair formation). All these processes are known to be regulated by ABA in higher plants, but ABA levels were unaffected in roots and leaves of NatA-depleted plants. The latter strongly argues against the activation of ABA synthesis or decreased degradation of ABA as molecular trigger for constitutive induction of drought stress response in NatA-depleted plants. The significant overlap between ABA-mediated processes and the response to NatA depletion in combination with the decreasing effect of ABA on NatA activity in the wild type suggests that NatA acts downstream of ABA during the induction of the drought stress response. This could be either achieved by altered biological activity of many NatA substrates in different pathways or by modified activity of a few key regulators of the drought response. Indeed, many drought stress-responsive transcription factors are regulated by ubiquitination-dependent proteasomal degradation^20^. In humans, NAA10 is a negative regulator of the proteasome^33^, which is in agreement with the significant upregulation of the ubiquitination-mediated proteolysis pathway in NatA-depleted plants. Furthermore, degradation of proteins by the Ac/N end rule pathway that accepts acetylated N termini starting with A, V, S, T and C^6^ could be perturbed in the plant NatA mutants. The unacetylated protein N termini might earmark these proteins for different degradation routes in plants. A similar scenario has been evidenced for proteins starting with iMet in yeast *natC* mutants^8^.

In summary, our findings demonstrate that imprinting of the proteome by the NatA complex is an evolutionary conserved process in higher eukaryotes and is dynamic in higher plants. We propose that the regulation of NatA by ABA triggers the response of plants towards drought stress by regulation of global transcription and development (root morphology). Consequently, NatA-depleted plants are preadapted and highly drought resistant. Thus, we propose that N^α^-terminal acetylation is a general cellular surveillance mechanism in higher plants that contributes significantly to the response to abiotic stresses. These findings suggest new targets to genetically engineer plants that are more resistant towards water limitation, an important trait for crop yield.

## Methods

### Plant material and growth conditions

All work was performed with *Arabidopsis thaliana* ecotype Col-0. One T-DNA insertion line for *AtNAA10*, naa*10*-*1* (Wisc DsLOX289_292G3), *AtNAA20*, *naa20-1* (SALK_027687) and two T-DNA insertion lines for *AtNAA15*, naa*15*-*1 (SAIL_812_B10)* and *naa15*-*2* (Wisc DsLOX481_484J20) were obtained from the SAIL, SALK and Wisconsin collection, respectively. Transformation of *Agrobacterium tumefaciens* C58 with binary vectors and subsequent transformation and selection of *Arabidopsis thalina* cv Col-0 was carried out as described in ref. 34. Heterozygous T-DNA lines for NAA10 and NAA15, homozygous T-DNA lines for NAA20, wild-type and *Arabidopsis* plants expressing amiRNA-Naa10 and amiRNA-Naa15 (F2 or F3-generation) were grown in climate chambers in growth medium containing one half soil and one half substrate 2 (Klasmann-Deilmann, Germany) under controlled conditions: 8.5 h light, 100 μE light photon flux density, 24 °C at day, 18 °C at night and 50% humidity. Rosette leaves of 8-week-old complemented *naa10-1* plants were pooled before fresh weight determination.

### PCR

PCR for identification of T-DNA insertion lines was performed with the Taq-DNA Polymerase from New England Biolabs (M0267L). Genotyping of T-DNA insertion lines *naa10-1*, *naa15-1*, *naa15-2* and *naa20-1* was conducted with specific primer combinations for the wild-type allele (NAA10_LP, NAA10 RP, NAA15_1_LP, NAA15_1_RP, NAA15_2_LP, NAA15_2_RP, NAA20_1_LP, NAA20_1_RP) and mutant allele (Wisc_BP, SAIL_BP, SALK_BP). For cloning, DNA was amplified with the high-fidelity DNA polymerase Phusion (New England Biolabs, M0530L). In both cases, the PCR reactions were performed according to the supplier's instructions manual. Sequences of the respective primers are provided in Supplementary Table 1.

### Quantitative real-time PCR

Total RNA from leaf tissue was extracted with the RNeasy Plant Kit (Qiagen, Germany) according to the manufacturer's protocol. Total RNA was transcribed into complementary DNA (cDNA) and analysed by qRT–PCR as described in ref. 35. For quantification of artificial microRNA (amiRNA) in total RNA samples, the amiRNA was polyadenylated, followed by the addition of a universal tag and reverse transcription using the miScript II RT Kit (Qiagen, Germany). The resulting cDNA was analysed by qRT–PCR as described above using actin_7 as reference. The amiNaa10_f and the miScript universal tag primer (supplied with the miScript SYBR II RT Kit, Qiagen) served as primers for specific amplification of amiRNA against *NAA10*. The sequences of primers used for qRT–PCR are depicted in Supplementary Table 1.

### Generation of NAA10 and NAA15 antibodies

The full-length *AtNAA10* was PCR amplified and cloned into pET32a (TRX(Thioredoxin)/His-fusion) using BamHI and XhoI restriction sites. A fragment of *AtNAA15* including the amino-acid residues 575–668 was PCR amplified and cloned into pET20b (His-fusion) using the XhoI and BamHI restriction sites. Oligospecific primers used to amplify *AtNAA10* and *AtNAA15*: NAA10_BamHI_f, NAA10_XhoI_r, NAA15_BamHI_f and NAA15_XhoI_r. Correct cloning was verified by DNA sequencing. The primer sequences are listed in Supplementary Table 1. The pET32a-AtNAA10 and pET20b-AtNAA15 plasmids were transformed into *E. coli* HMS174 (DE3) cells (Invitrogen) by electroporation. Cell cultures (300 ml) were grown in LB (lysogeny broth) medium to an OD_600 nm_ of 0.8 at 37 °C and protein expression was induced by 1 mM isopropyl-β-D-thiogalactoside. After 5 h of incubation, the cultures were harvested by centrifugation and the pellets stored at −80 °C. The purification was performed as described for pETM41-AtNAA10. The purified antigen was used for the immunization of rabbits.

### Constructs for subcellular localization and complementation

For localization*, AtNAA10* and *AtNAA15* were PCR amplified by using the NAA10_L_f, NAA10_L_r, NAA15_L_f and NAA15_L_r primers, and cloned via Gateway technology (Invitrogen) in the binary vector pK7YWG2.0, which contains the coding sequence for YFP (yellow fluorescent protein). YFP-fusion proteins were expressed in tobacco cells 2 days after transient transformation with the respective pK7YWG2.0 construct. Fluorescence was analysed by confocal laser scanning microscopy^36^. *Nicotiana tabacum* cells were transiently transformed^37^. For complementation, *AtNAA10* was amplified using the primers NAA10_C_f and NAA10_C_r and cloned into the binary expression vector pK2GW7 by using the Gateway technology (Invitrogen). Correct cloning was verified by DNA sequencing. Primer sequences are listed in Supplementary Table 1.

### Constructs for expression in mammalian and *E. coli* cells

*AtNAA10* and *AtNAA15* were PCR amplified from pDONR201/AtNAA10 and pDONR201/AtNAA15 and cloned into pcDNA3.1-V5 vector and pcDNA3.1 His Max vector (Invitrogen), respectively, for expression in mammalian cells. The oligospecific primers AtNAA10_f, AtNAA10_r, AtNAA15_f and AtNAA15_stop_r were used to amplify *AtNAA10* and *AtNAA15*. For *E. coli* expression, *AtNAA10* was PCR amplified and cloned into pETM-41 (MBP (maltose-binding protein)/His-fusion) using the Acc65I and NcoI restriction sites of the vector and BsmBI cutting of the *AtNAA10* fragment. AtNAA10_BsmBI_f and AtNAA10_BsmBI_r primers were used to ampltfy *AtNAA10*. pETM-41 was generously provided by G. Stier, EMBL, Heidelberg, Germany. For all constructs, correct cloning was verified by DNA sequencing. Primer sequences are provided in Supplementary Table 1.

### Expression and purification

The pETM-41-AtNAA10 plasmid was transformed into *E. coli* BL21 Star (DE3) cells (Invitrogen) by heat shock. Cell culture (200 ml) was grown in LB (lysogeny broth) medium to an OD_600 nm_ of 0.6 at 37 °C and subsequently transferred to 20 °C. After 30 min of incubation, protein expression was induced by isopropyl-β-D-thiogalactoside (1 mM). After 17 h of incubation, the cultures were harvested by centrifugation and the pellets stored at −20 °C. *E. coli* pellets containing recombinant proteins were thawed at 4 °C and the cells lysed by sonication and french press in lysis buffer (1 mM DTT, 50 mM Tris-HCl (pH 7.4), 300 mM NaCl, 1 tablet EDTA-free protease Inhibitor cocktail per 50 ml (Roche)). The cell extracts were applied on a metal affinity FPLC column (HisTrap HP, GE Healthcare, Uppsala, Sweden). Appropriate fractions containing recombinant protein MBP-His-AtNAA10) were pooled and further purified using size-exclusion chromatography (Superdex 75, GE Healthcare). The purity of the fractions corresponding to purified monomeric recombinant proteins were analysed on Coomassie-stained SDS–polyacrylamide gel electrophoresis (SDS–PAGE) gels and the protein concentrations determined by OD_280 nm_ measurements.

### *In vitro* Nats assay

Purified MBP-His-AtNAA10 (0.5 μM) was mixed with selected oligopeptide substrates (200 μM) and 300 μM of acetyl-CoA in a total volume of 60 μl acetylation buffer. The samples were incubated at 37 °C for 60 min. The enzyme activities were quenched by adding 5 μl of 10% TFA. The acetylation reactions were quantified using RP-HPLC^38^. Synthetic peptides were custom made (Biogenes) to a purity of 80–95%. All peptides contain seven unique amino acids at their N terminus, as these are the major determinants influencing NTA. The next 17 amino acids are essentially identical to the ACTH peptide sequence (RWGRPVGRRRRPVRVYP) except that the lysines were replaced by arginines to minimize any potential interference by N^ɛ^-acetylation. Peptide sequences: STPD-, SMCA4 (P51532): [H]STPDPPLRWGRPVGRRRRPVRVYP[OH]; SPTP-, THO complex subunit 1 (Q96FV9): [H]SPTPPLFRWGRPVGRRRRPVRVYP[OH]; MELL-, MyoD (P15172): [H] MELLSPPRWGRPVGRRRRPVRVYP [OH].

### Immunoprecipitation

HEK293 cells were co-transfected with plasmids encoding Xpress-AtNAA15 and AtNAA10-V5 using FuGENE6 (Roche), harvested 48 h post transfection and lysed in 1 ml IPH buffer (50 mM Tris-HCl pH 8.0, 150 mM NaCl, 0.5% NP-40, 5 mM EDTA, 1 mM Na_3_VO_4_, 2 mM pefabloc and 1 × complete protease inhibitor cocktail (Roche)). Approximately 1 × 10^7^ cells were used for each sample. Protein A/G Plus-Agarose slurry (50 μl, Santa Cruz Biotechnology, Santa Cruz, USA) was added and incubated for 1 h at 4 °C. After centrifugation at 500*g* for 5 min, the supernatants were incubated with 3.0 μg anti-V5 mouse monoclonal IgG_2a_ ab (Invitrogen), anti-Xpress mouse monoclonal IgG_1_ ab (Invitrogen) or unspecific IgG_2a_ ab (DAKO) and incubated for 4 h at 4 °C. Later, 70 μl of Protein A/G Plus-Agarose was added and incubated for 16 h at 4 °C with the samples. The immunoprecipitate samples were collected by centrifugation at 500*g* for 5 min, washed four times with 1 × PBS.

### SDS–PAGE and immunological detection

Soluble protein extracts were subjected to discontinuous SDS–PAGE in Mini-ProteanTM II cells (BioRad)^39^ and immunoblotting. For the immunoprecipitation assay, the anti-V5, anti-Xpress and horseradish peroxidase-linked anti-mouse antibodies (Amersham) were diluted 20,000-fold in 1 × PBS (10 mM Na_2_HPO_4_, 1.8 mM KH_2_PO_4_, 137 mM NaCl, 2.7 mM KCl) before usage. The specific antibodies against OAS-TLs, the ribosomal protein S14 (#AS12 2,111, Agrisera) and NAA15 were diluted 1,000, 8,000 and 8,000-fold in TBS-T (50 mM Tris pH 7.6, 150 mM NaCl, 0.05% Tween-20) for quantification of respective proteins in fractions of differential ultra-centrifuged soluble leaf proteins. The abundance of NAA10 and NAA15 in soluble leaf proteins isolated from wild-type, amiNaa10 and amiNaa15 plants were determined with αNAA10 diluted 5,000-fold or αNAA15 diluted 8,000-fold in TBS-T. The horseradish peroxidase-linked anti-rabbit antibody (#AS10 852, Agrisera) was diluted 25,000-fold in TBS-T. Membranes were developed using SuperSignal West Dura Extended Duration Substrate (Thermo Scientific) according to the manufacturer's protocol. The resulting signals were recorded using the ImageQuant LAS 4,000 (GE Healthcare) and subsequently quantified with the ImageQuant TL Software (GE Healthcare). The Original images of the cropped gels/blots shown in the paper are depicted in Supplementary Fig. 7.

### BiFC assay

For construction of BiFC vectors, *AtNaa10* and *AtNaa15* were PCR amplified from total cDNA and cloned into pPCR-Script (Stratagene) before being subcloned into the final destination vectors (pWEN-NY or pWEN-CY). For construction of the pWEN-NY or pWEN-CY vectors^40^, *AtNAA10* was amplified with NAA10_B_f and NAA10_B_r and *AtNAA15* with NAA15_B_f and NAA15_B_r. For all constructs, correct cloning was verified by DNA sequencing. Constructs were transfected into tobacco leaf cells by particle bombardment^41^ and samples analysed after 24–48 h using a Nikon A1R confocal laser microscope. As negative controls, the expression of the NY and CY fragments in combination with *AtNAA* cDNAs fused to -CY or -NY respectively was analysed and did not produce detectable fluorescence. All combinations were repeated in triplicate.

### Yeast two-hybrid assays

For construction of yeast two-hybrid vectors, *AtNaa10* and *AtNaa15* were PCR amplified from total cDNA and cloned into pPCR-Script (Stratagene) before being subcloned into the final destination vectors (pGBKT7 and pGADT7). For construction of the pGBKT7 and pGADT7 vectors (Clontech), *AtNAA10* was amplified with the oligospecific primers NAA10_Y_f and NAA10_Y_r and *AtNAA15* (1-1236 base pairs) was amplified with NAA15_Y_f and NAA15_Y_r. Primer sequences are listed in Supplementary Table 1. For all constructs, correct cloning was verified by DNA sequencing. Yeast HF7c cells were co-transformed with combinations of pGADT7 and pGBKT7 vectors according to the manufacturer's instructions (Clontech). After 3–4 days of incubation at 30 °C, single colonies were inoculated in synthetic dropout (SD) media lacking leucine and tryptophan (SD–TL) and grown at 30 °C in a shaking incubator for one night. Cultures were adjusted to OD_600_ of 0.01 and 5 μl of each dilution spotted onto SD–TL plates or plates lacking leucine, tryptophan and histidine (SD–HTL) and containing 0.01 mM 3-AT (3 amino-1,2,4-triazole) to monitor *HIS3* reporter expression by cell growth. Plates were incubated at 30 °C for 2 days, and images were captured before each spot was resuspended in SD–HTL media and the OD_600_ measured. The ratio of growth on SD–TL and SD–HLT+3-AT was calculated for three individual pairs of spots and the average and s.d. of these ratios calculated.

### Differential ultracentrifugation

Total protein was extracted from 10 day-old in-flow culture under continuous light grown seedlings (growth medium contained 2.2 g l^−1^ MS-salts (Duchefa), 1% (w/v) Sucrose, 0.4 g l^−1^ MES, 0.1% microagar; pH 5.9). Frozen and grinded seedling material (300 mg) was resuspended in 1 ml lysis buffer (50 mM HEPES KOH pH 7.4, 50 mM KCl, 25 mM MgCl_2_, 5 mM EGTA, 15.4 U ml^−1^ heparin, 18 μM cycloheximide, 15.5 μM chloramphenicol, 2% Triton-X-100, 2% Brij 35, 2% Tween-40, 2% NP-40, 0.5 mM phenylmethylsulphonyl fluoride). The resulting supernatant was loaded onto a linear 10–50% sucrose gradient in 20 mM HEPES KOH pH 7.4, 20 mM KCl and 10 mM MgCl_2_ and centrifuged in a SW40 rotor at 35,000 r.p.m. for 2.5 h at 4 °C. Polysome profiles were obtained by measuring absorbance at 254 nm. The obtained protein fractions were pooled into nine groups in which the abundance of AtNAA15, the ribosomal protein S14 and the cytosolic, plastidic as well as mitochondrial isoforms of the *O*-acetylserine(thiol)lyase (OAS-TL) was quantified by immunological detection using specific antisera (see section: SDS–PAGE and immunological detection).

### Differential interference contrast microscopy

Development of embryos in wild-type and transgenic plants were analysed by differential interference contrast microscopy. Carpels were removed from *Arabidopsis* siliques and the embryos were fixed in Hoyer's solution (7.5 g gum arabic, 100 g chloral hydrate, 5 ml glycerol, 60 ml water) for at least 16 h at room temperature. Images were recorded with the Leica DMIRB microscope at 20-fold magnification (Objective: N Plan L × 20/0.40 CORR).

### amiRNAi constructs against NAA10 and NAA15

The amiRNAs against *AtNAA10* and *AtNAA15* were designed with the WMD 2-Web MicroRNA Designer (www.weigelworld.org). The amiRNA of NAA10 was constructed by using an overlapping PCR approach with the primers NAA10P1, NAA10P2, NAA10P3, NAA10P4, pRS300-A and pRS300-B according to ref. 42, which resulted in amplification of the amiRNA loop structure, the NAA10-specific sequence and terminal restriction sited for KpnI and BamHI at the 5′ and 3′end, respectively. The resulting PCR product was digested with KpnI and BamHI and cloned into the plant transformation vector pBinAR under the control of the CaMV35S-promotor of the cauliflower mosaic virus resulting in pBinAR-amiNaa10. The same strategy was applied to NAA15 using the primers NAA15P1, NAA15P2, NAA15P3, NAA15P4, pRS300-A and pRS300-B and resulted in the construction of pBINAR-amiNaa15. The primer sequences are listed in the Supplementary Table 1.

### Quantification of ABA in *Arabidopsis thaliana*

For phytohormone analysis, finely ground leaf or root material (60–150 mg fresh frozen) was extracted with 1 ml of methanol containing 40 ng D6-ABA (Santa Cruz Biotechnology) as internal standard. The homogenate was mixed for 30 min and centrifuged at 20,000*g* for 20 min at 4 °C. The supernatant was collected. The homogenate was re-extracted with 500 μl methanol, mixed and centrifuged and supernatants were pooled. The combined extracts were evaporated in speed-vac at 30 °C and redissolved in 500 μl methanol. Chromatography was performed on an Agilent 1,200 HPLC system (Agilent Technologies). Separation was achieved on a Zorbax Eclipse XDB-C18 column (50 × 4.6 mm, 1.8 μm, Agilent). Formic acid (0.05%) in water and acetonitrile were employed as mobile phases A and B, respectively. The elution profile was: 0–0.5 min, 5% B; 0.5–9.5 min, 5–42% B; 9.5–9.51 min 42–100% B; 9.51–12 min 100% B and 12.1–15 min 5% B. The mobile phase flow rate was 1.1 ml min^−1^. The column temperature was maintained at 25 °C. An API 5,000 tandem mass spectrometer (Applied Biosystems) equipped with a Turbospray ion source was operated in negative ionization mode. The instrument parameters were optimized by infusion experiments with pure standards, where available. The ionspray voltage was maintained at at −4,500 eV. The turbo gas temperature was set at 700 °C. Nebulizing gas was set at 60 p.s.i., curtain gas at 25 p.s.i., heating gas at 60 p.s.i. and collision gas at 7 p.s.i. Multiple reaction monitoring was used to monitor analyte parent ion → product ion: *m/z* 263.0→153.2 (CE −22 V; DP −35 V) for ABA; *m/z* 269.0→159.2 (CE −22 V; DP −35 V) for D6-ABA. Both Q1 and Q3 quadrupoles were maintained at unit resolution. Analyst 1.5 software (Applied Biosystems) was used for data acquisition and processing. Linearity in ionization efficiencies were verified by analysing dilution series of a standard. ABA was quantified relative to the signal of the internal standard.

### Quantification of N-terminal protein acetylation

Liquid nitrogen-frozen *Arabidopsis* plant tissues were grounded in 2 ml microcentrifuge tubes containing 3 and 5 mm iron beads for 1 min each, using a MM 300 mixer mill at 30 Hz (Qiagen). The resulting fine powder was dissolved in 1 ml of lysis buffer (buffer D^43^). The homogenates were incubated at 4 °C for 30 min with shaking. The supernatants were separated from the insoluble fraction by centrifugation at 15,000*g* at 4 °C for 30 min and used to determine protein concentration using the Bradford protocol. As previously described^44^, 1 mg of proteins was denaturated in 6 M Guanidine-HCl, 50 mM Tris-HCl (pH 8) and 4 mM DTT, reduced for 15 min at 95 °C and finally alkylated by the addition of iodacetamide (55 mM) for 1 h at room temperature. Proteins were precipitated by the addition of four times the sample volume of cold acetone followed by 1 h centrifugation at −20 °C. The resulting pellet was resuspended in 50 mM phosphate buffer (pH 7.5) and was subjected to chemical acetylation of the free N terminus amino groups with N-acetoxy-[^2^H_3_]-succinimide according to ref. 45. After 90 min incubation at 30 °C, potential *O*-acetylation of Ser, Thr and Tyr side chains were reversed by adding 10 μl of 50% of hydroxylamine and incubated for 20 min at room temperature. The sample was acetone cold precipitated to remove chemical reagent before trypsin digestion by the addition of 1/100th (w/w) of TPCK-treated bovine trypsin (Sigma-Aldrich) for 1 h at 37 °C twice. Peptides were desalted by Sep-Pak (Waters, Milford, MA) solid phase extraction as recommended by the supplier. The retained material was eluted with 80% ACN, 0.1% TFA followed by evaporation to dryness. The peptide mixture was resuspended in SCX-LC buffer A (5 mM KH_2_PO_4_, 30% ACN and 0.05% formic acid) and loaded on a Summit LC system (Dionex, Sunnyvale, CA) equipped with Polysulfoethyl A 200 × 2.1 mm 5 μm 200 Å column (PolyLC, Colombia, MD). Peptides were eluted with a gradient of increasing KCl (SCX-LC Buffer B: 350 mM KCl in SCX-LC Buffer A; 0–5 min, 0% B; 15–40 min, 5–26% B; 40–45 min, 26–35% B). Fractions were collected every 2 min for 50 min followed by evaporation to dryness and stored at −20 °C before nano-LC-MS analysis.

Fractions eluted from SCX chromatography with retention time between 2 and 10 min (fractions 2–5) and 10 and 22 min (Fraction 6–11) were resuspended in 25 and 30 μl (nLC Buffer A: 0.1% FA, 5% ACN) respectively. Each fraction was analysed individually (1 h cycle acquisition method) and 15 μl were loaded onto a pre-column (NS-MP-10, Nanoseparation, Nieuwkoop, Netherland) at a maximum pressure of 200 bars of nLC buffer A followed by a separation using a Nikkyo Technos capillary column (NTCC-360/100-5-153, Nikkyo Technos Co., Tokyo, Japan) over a 42 min gradient (nLC buffer B: 0.1% formic acid in AcN; 5–35% B in 42 min) at a flow rate of 300 nl min^−1^. The previous fractions 2–5, 6–8 and 9–11 were combined together and 18 μl of this mixture was analysed twice on a 2 h cycle acquisition (5–35% nlC buffer B in 90 min). The first acquisition includes all precursor ion charges whereas the second acquisition reject singly charged precursors. The nano-LC is coupled to an Orbitrap Velos (Thermo scientific). The survey scan was acquired by Fourier-Transform MS scanning 400–2,000 Da at 30,000 resolution using internal calibration. The 20 most intense ions were subject to high collision dissociation MS with 20 s exclusion time for the selected precursor. MS/MS spectra with parent ion signal higher than one count and with a signal over noise ratio higher than 1.5 are extracted with Proteome Discoverer (Thermo Scientific, Ver. 1.4) and submitted to Mascot 2.4 software used for protein identification and co/post-translational modification characterization using the *Arabidopsis* Information Resource (TAIR) database for *A. thaliana* (TAIR ver. 10, www. arabidopsis.org)^46^. Trypsin/P rule was used with parent and fragment mass tolerance defined as 10 p.p.m. and 0.7, respectively. Carbamidomethylcysteine and d3-acetyl on Lys were considered as fix modifications, whereas Met-oxidation, Lys acetylation, protein NTA and d3-NTA were considered as variable modifications. All data were filtered at 1% protein false discovery rate and only peptides with score higher than 30 are retained for the final data treatment. To extract specifically N-terminal peptides, Mascot searches were exported in XML format and submitted to an in-house script written in python. The parsing function searched for modifications and collected peptides with defined modifications such as d0/d3-NTA. If the same peptide was identified several times with the same modification, irrespectively of the peptide charge, only the highest score was retained for further investigation.

Quantification for N terminus peptides required the aid of Mascot Distiller (Ver. 2.5.1, Matrix Science). Extraction parameters were optimized for the OrbiTrap acquisition files with a minimum S/N of 1, precursor charges considered 1–5, Corr. Thr. >0.7 and no grouping assignments. Mascot distiller requests for protein identification were performed using the Mascot identification tool with the previously defined searching parameters including additionally the quantitation method associated with d0/d3-NTA ratio that provides finally the NTA yield. Since Mascot Distiller is designed to deliver a quantification value for the whole protein including all the characterized peptides and not the protein N terminus only, we used a Python script able to parse the Mascot Distiller export files in xml format to recalculate the NTA yield. Filtering parameters used by Mascot Distiller were also used in the extraction script, that is, E Val.<0.1, Std. Err.<0.1, Mascot Score > 30, Corr. > 0.5, Fraction > 0.5, Sc. *P*> 0.3. NTA yield was determined from the d0/d3 ratio and expressed in% of NTA for the N termini.

### Determination of the global transcriptome

Total RNA was extracted from leaf tissue from wild-type, amiNaa10 and amiNaa15 plants grown for 52 days on soil under short-day conditions and from wild-type plants grown for 42 days on soil under short-day conditions followed by 10 days of drought using the peqGOLD Total RNA Kits (Peqlab) according to the manufacturer's protocol. Total RNA was prepared using Trizol (Gibco) followed by additional purification using the RNeasy Mini Kit (Qiagen). RNA was tested by capillary electrophoresis on an Agilent 2,100 bioanalyzer (Agilent) and high quality was confirmed

Gene expression profiling was performed using arrays of Aragene-1_0-st-type from Affymetrix. Biotinylated antisense cRNA was then prepared according to the Affymetrix standard labelling protocol. Later, the hybridization on the chip was performed on a GeneChip Hybridization oven 640, then dyed in the GeneChip Fluidics Station 450 and thereafter scanned with a GeneChip Scanner 3,000. All of the equipment used was from the Affymetrix-Company (Affymetrix, High Wycombe, UK).

A Custom CDF Version 16 with TAIR-based gene definitions was used to annotate the arrays^47^. The Raw fluorescence intensity values were normalized applying quantile normalization and RMA background correction. Analysis of variance was performed to identify differential expressed genes using a commercial software package SAS JMP10 Genomics, version 6, from SAS (SAS Institute, Cary, NC, USA). A false positive rate of *a*=0.05 with false discovery rate correction was taken as the level of significance.

The over-representation analysis is a microarray data analysis that uses predefined gene sets to identify a significant over-representation of genes in data sets^48^. Pathways belonging to various cell functions such as cell cycle or apoptosis were obtained from public external databases (KEGG, http://www.genome.jp/kegg/ and GO, http://www.geneontology.org/). The analysis was performed using the DAVID Bioinformatics Resources 6.7 (ref. 49).

### Determination of free N termini

To quantify the free N termini level, soluble proteins were extracted from leaves in 50 mM sodium citrate buffer containing 1 mM EDTA (pH 7.0) and desalted using PD SpinTrap G-25 columns (GE Healthcare) to remove free amino acids. Crude protein extract (2.5 μM) was incubated with 0.5 mM NBD-Cl (Sigma-Aldrich) in 50 mM sodium citrate buffer containing 1 mM EDTA (pH 7.0) for 14 h at room temperature. The fluorescence intensity was quantified with a FLUOstar Omega plate reader (BMG Labtech; excitation: 470±10; emission: 520 nm). For the quantification of free N termini in response to ABA (Sigma-Aldrich), leaf discs were floated (30 mg) in ½ × Hoagland medium supplemented with 50 μM ABA for 2, 4, 6 and 8 h before analysis.

### Determination of ROS in intact guard cells

ROS accumulation in guard cells was quantified using the H_2_O_2_-specific dye H_2_DCF-DA (Life Technologies). Epidermal peels of wild-type, amiNaa10 and amiNaa15 plants were floated for 120 min on 30 mM KCl, 10 mM MES (pH 6.15). H_2_O_2_ was stained for 10 min with 50 μM H_2_DCF-DA, which was added to the medium. To analyse the impact of ABA treated on ROS production, peels were preincubated with 50 μM ABA for 20 min before H_2_O_2_ staining. Control samples were treated with 0.1% ethanol. The H_2_O_2_-specific fluorescence was recorded in the guard cells with the C2 plus confocal laser microscope (Nikon; excitation 488 nm; emission 525 nm) and evaluated with the Fiji image-processing package.

### Determination of stomata density

For determination of the stomata density, imprints of the abaxial leaf side of 5-week-old wild-type, amiNaa10 and amiNaa15 plants were created on glass microscope slides using super glue and analysed with the DMIRB microscope (Leica).

### Determination of stomatal aperture

Size of stomatal aperture was determined by imaging the abaxial leaf side of 5-week Fold wild-type, amiNaa10 and amiNaa15 plants with a confocal laser microscope (Zeiss LSM 510). Data analysis was performed with the Fiji image-processing package.

### Determination of primary root length and lateral root density

After growing for 5 weeks on ½ × Hoagland or for 14 days on 1 × MS solid medium (Duchefa), roots from wild-type, amiNaa10, amiNaa15 and *naa20* plants were photographed with a digital camera. The primary root length and the number of lateral roots were measured using the Fiji image-processing package.

### Determination of the transpiration rate

To measure leaf water loss, fully expanded leaves were detached from 5-week-old plants and placed abaxial side up in open petri dishes at room temperature. The weight of the leaves was monitored during 2 h with a precision balance (Sartorius).

### Quantification of drought stress

To characterize the drought stress, we determined the water content of the soil by subtraction of the weight of completely dried soil from the weight of the soil at day of analysis. The completely dried soil weight was determined by drying the respective soil for 4 days at 80 °C.

The RWC leaves was calculated by using the formula: RWC 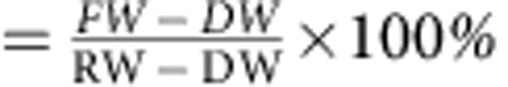. The fresh weight of rosette leaves (*N*=3) of individual plants (*N*=4) were measured. Subsequently, the leaves were rehydrated for 6 h in 4 °C cold water to determine the rehydrated weight of the sample. The dry weight was determined after drying of leaves for 18 h at 80 °C.

### Basic statistical analysis

Regression analyses of data sets were performed with SigmaPlot 12.0 that uses the Marquardt–Levenberg algorithm for determination of independent variables. Constant variance and normally distribution of data were carefully checked with SigmaPlot 12.0 before statistical analysis. Comparison of means from different sets of data was analysed for statistical significance with the unpaired *t*-test or the Mann–Whitney *U*-rank test, if data set was not normally distributed. Significant differences (*P*<0.05) are indicated by asterisks.

## Additional information

**Accession codes:** The mass spectrometry proteomics data are deposited in the PRIDE repository with the data set identifier PXD002069. The microarray data sets are uploaded to the NCBI GEO database under the GEO Accession number GSE65414.

**How to cite this article:** Linster, E. *et al*. Downregulation of N-terminal acetylation triggers ABA-mediated drought responses in *Arabidopsis*. *Nat. Commun.* 6:7640 doi: 10.1038/ncomms8640 (2015).

## Supplementary Material

Supplementary Information Supplementary Figures 1-7, Supplementary Table 1 and Supplementary References

Supplementary Data 1 List of all identified and quantified protein N-termini for NatA, Nat B, NatC and other unassigned substrates in wild type, amiNaa10 and amiNaa15 plants

## Figures and Tables

**Table 1:** Gene annotation enrichment analysis of transcripts regulated in NatA-depleted plants.

**Pathway**	**Annotation**	**Trend**	**Fold enriched**	***P*** **value**
Sulfate assimilation	P02778	Downregulated	17.45	0.01
Indole alkaloid biosynthesis	ATH00901	Downregulated	13.17	0.02
Flavonoid biosynthesis	ATH00941	Downregulated	12.94	0.00
α-Linolenic acid metabolism	ATH00592	Downregulated	7.52	0.00
Phenylpropanoid biosynthesis	ATH01061	Downregulated	2.13	0.01
Ubiquitin-mediated proteolysis	ATH04120	Upregulated	3.82	0.01

Wild-type and NatA-depleted plants (aminNaa10 and amiNaa15) were grown for 6 weeks under short-day condition with regular water supply. Transcripts were quantified with the Gene 1.0 ST Array (Affymetrix, Germany). Significantly regulated genes were defined by *P*<0.05 and >1.5-fold change when compared with wild type (*N*=4). Gene annotation analysis of these genes was performed with the DAVID Bioinformatics Resources tool v.6.7 (http://david.abcc.ncifcrf.gov).
