# Supplementary material for: Downregulation of N-terminal acetylation triggers ABA-mediated drought responses in Arabidopsis
Source: Nat Commun. 2015 Jul 17;6:7640. doi: 10.1038/ncomms8640 (PMC4530475; doi:10.1038/ncomms8640)
Supplement: Supplementary Information — Supplementary Figures 1-7, Supplementary Table 1 and Supplementary References [file ncomms8640-s1.pdf]

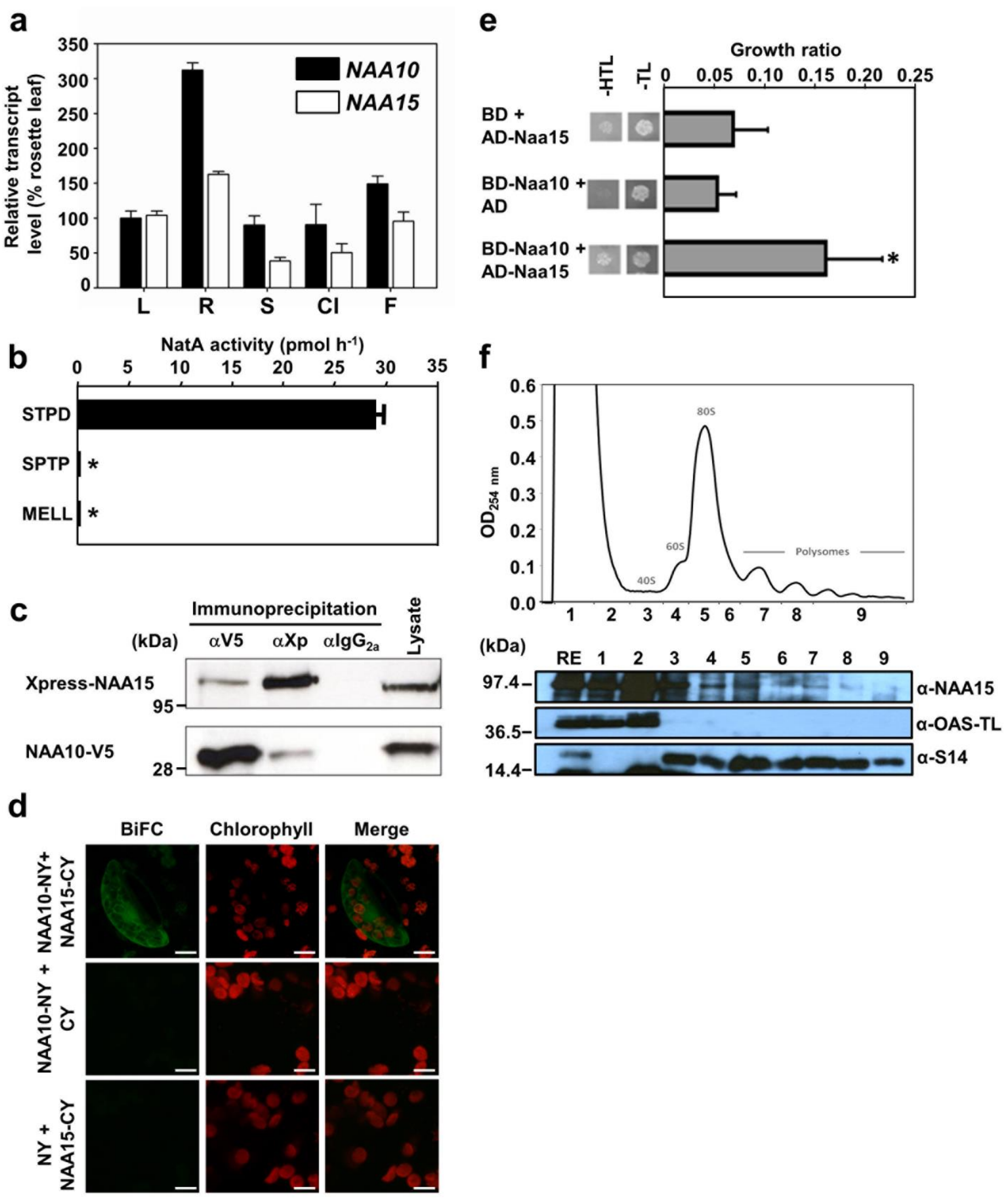

Supplementary Fig. 1: At5g13780 (NAA10) and At1g80410 (NAA15) form a complex with NatA type N-terminal protein acetylation activity

a) Transcript levels of *NAA10* (black) and *NAA15* (white) in leaves (L), root (R), shoot (S), cauline leaves (CL), and flowers (F) of wild type *Arabidopsis* as determined by qRT-PCR. Transcript levels in 6-week old rosette leaves were set to 100 % (N=3). **b**) N-terminal acetyltransferase activity of MBP-His-AtNAA10 was determined towards three 24-mer peptides that differ only in sequence of the seven N-terminal amino acid residues. The STPD peptide represents a known substrate of human Naa10p, the SPTP peptide represents an *in vivo* unacetylated protein serving as a negative control,

due to the inhibitory effect of the second proline residue. The MELL peptide is acetylated by human Naa20p/NatB. (N=3) **c**) Co-immunoprecipitation of NAA10-V5 and Xpress-NAA15 using antibodies against V5 and Xpress epitopes. **d**) Bimolecular fluorescence complementation assays were performed by co-expressing the full-length AtNAA10 fused to the N-terminal half of YFP (NY) and At NAA15 fused to the C-terminal half of YFP (CY) in *N. tabacum*. Samples were analyzed after 24-48 hours and fluorescence of the reconstituted YFP fluorophore was detected by confocal microscopy [1]. Negative controls were performed by expressing NAA10-NY with CY alone or NAA15-CY with NY alone. The auto-fluorescence of plastids is shown in red color. (Scale bar = 10 µm) **e**) The interaction of full-length NAA10 fused to the Gal4 DNA-binding domain (BD) with NAA15 (amino acids 1-412) fused to the Gal4 activation domain (AD) was assayed in yeast HF7c cells. The growth of yeast on −HTL media containing 0.01 mM 3-AT and on −TL media was assayed and the ratio of growth calculated as an indicator of the strength of interaction. Only yeast cells containing BD-NAA10 and AD-NAA15 were able to restore His auxotrophy (N=6). **f**) Soluble proteins of 10-days old seedlings were fractionated by differential-ultracentrifugation. The separation of mono- and polysomes from other soluble proteins was scanned on-line by determination of absorbance at 254 nm and revealed typical separation profile for ribosomes in plant seedlings. The presence of NAA15, S14 (a ribsome marker protein) and the mitochondrial (upper), plastidic (middle) and cytosolic *O*-acetylserine(thiol)lyase (lower signal) in the resolved fractions. **a, b, e**) Data are represented as mean ± standard deviations. Asterisks indicate significant differences ($p < 0.001$, Student's t-test).

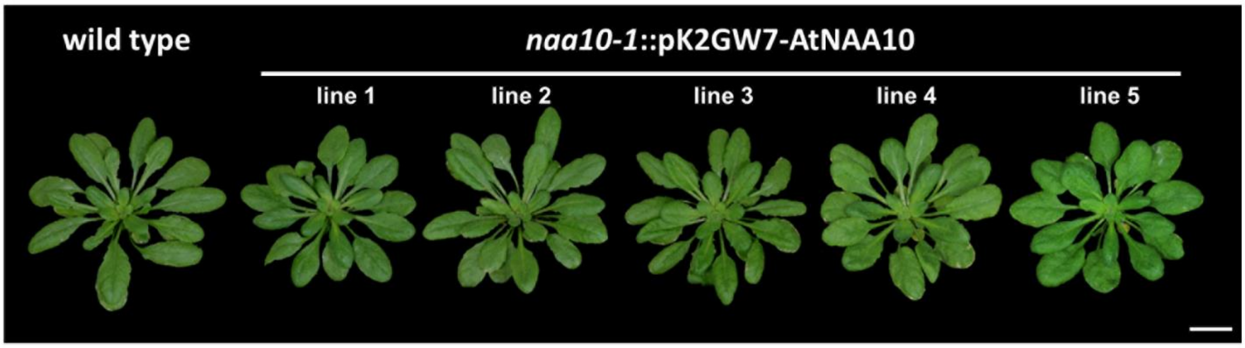

Supplementary Fig. 2: Genetic complementation of *naa10-1* plants with AtNAA10

Phenotype of 8-week old soil grown wild type and 5 homozygous *naa10-1* plant lines that were independently transformed with pK2GW7-AtNAA10. Transformation with pK2GW7-AtNAA10 results in ectopic expression of full length AtNAA10 under the control of the CaMV35S promotor..(Scale bar 2 cm)

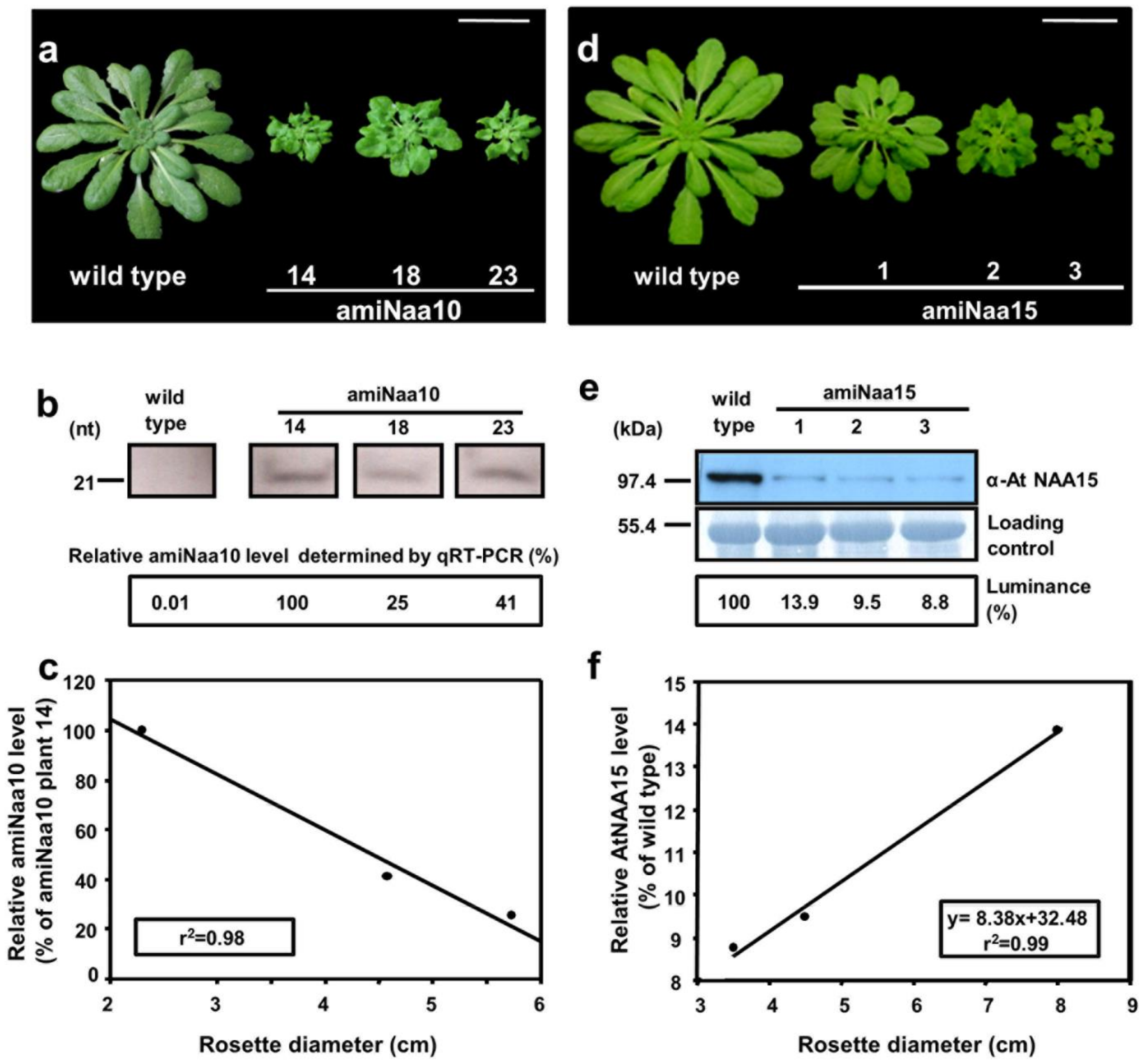

Supplementary Fig. 3: Quantification of NatA depletion in amiNaa10 and amiNaa15 plants

a) Growth phenotype of amiNaa10 plants grown for 7 weeks on soil under short day conditions. (scale bar = 4 cm) **b**) Quantification of artificial microRNA against *NAA10* (21 nucleotides) in amiNaa10 plants by northern hybridization and qRT-PCR. The transcript level of artificial microRNA against *NAA10* in line 14 of amiNaa10 has been set to 100 % for correlation analysis. **c)** Correlation analysis between abundance of artificial microRNA against *NAA10* and the degree of growth retardation in amiNaa10 plants. Data were obtained from analysis of plants shown in **a** and **b**. **d**) Growth phenotype of amiNaa15 plants grown for 7 weeks on soil under short day conditions. (scale bar = 4 cm). **e**) Immunological detection of NAA15 in amiNaa15 using a specific antiserum. **f)** Correlation analysis between abundance of NAA15 and the degree of growth retardation in amiNaa15 plants. Data were obtained from analysis of plants shown in **d** and **e**. Data in **c** and **f** were plotted and fitted using the enzyme kinetic module of Sigma Plot v 8.0 with a linear kinetic, r^2 = coefficient of determination.

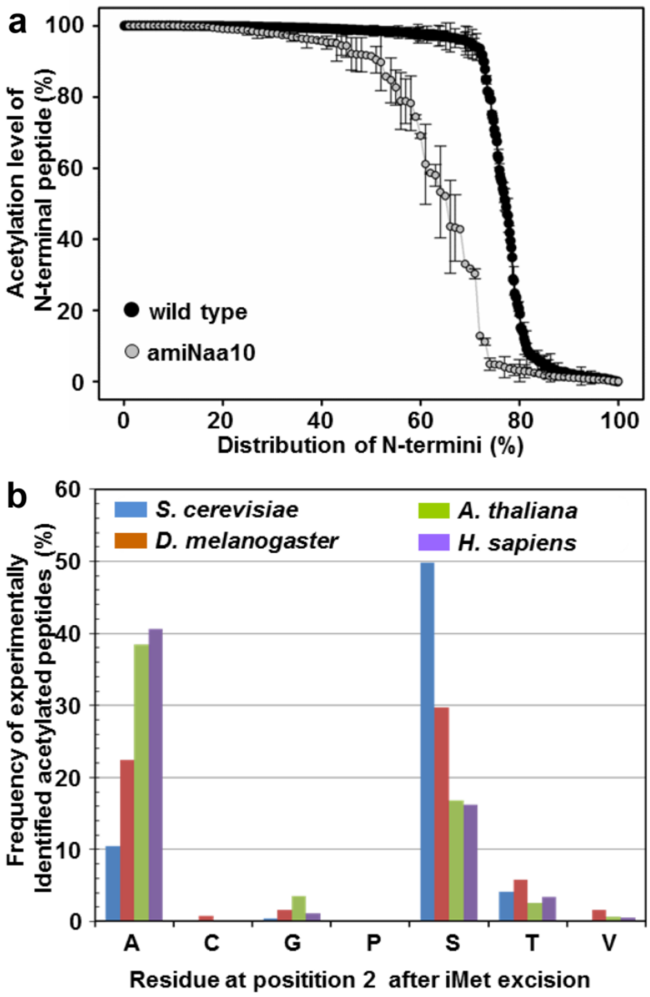

Supplementary Fig. 4: Decrease of acetylation in amiNaa10 and frequency of residues at position 2 from proteins that are subject to iMet removal in different organisms

a) Acetylation level of identified N-terminal peptides from soluble proteins extracted from leaves of 7-week old wild type (black) and amiNaa10 line 23 (grey) plants. After digestion of proteins N-terminal peptides were enriched by SCX-approach for MS-MS analysis. Data are represented as mean ± standard deviations (N = 4). **b**) Frequency of experimentally identified acetylated peptides that were subject of iMet removal (NatA substrates) in *Arabidopsis thaliana* (this study), *S. cerevisiae* [2], *D. melanogaster* and *H. sapiens* [3].

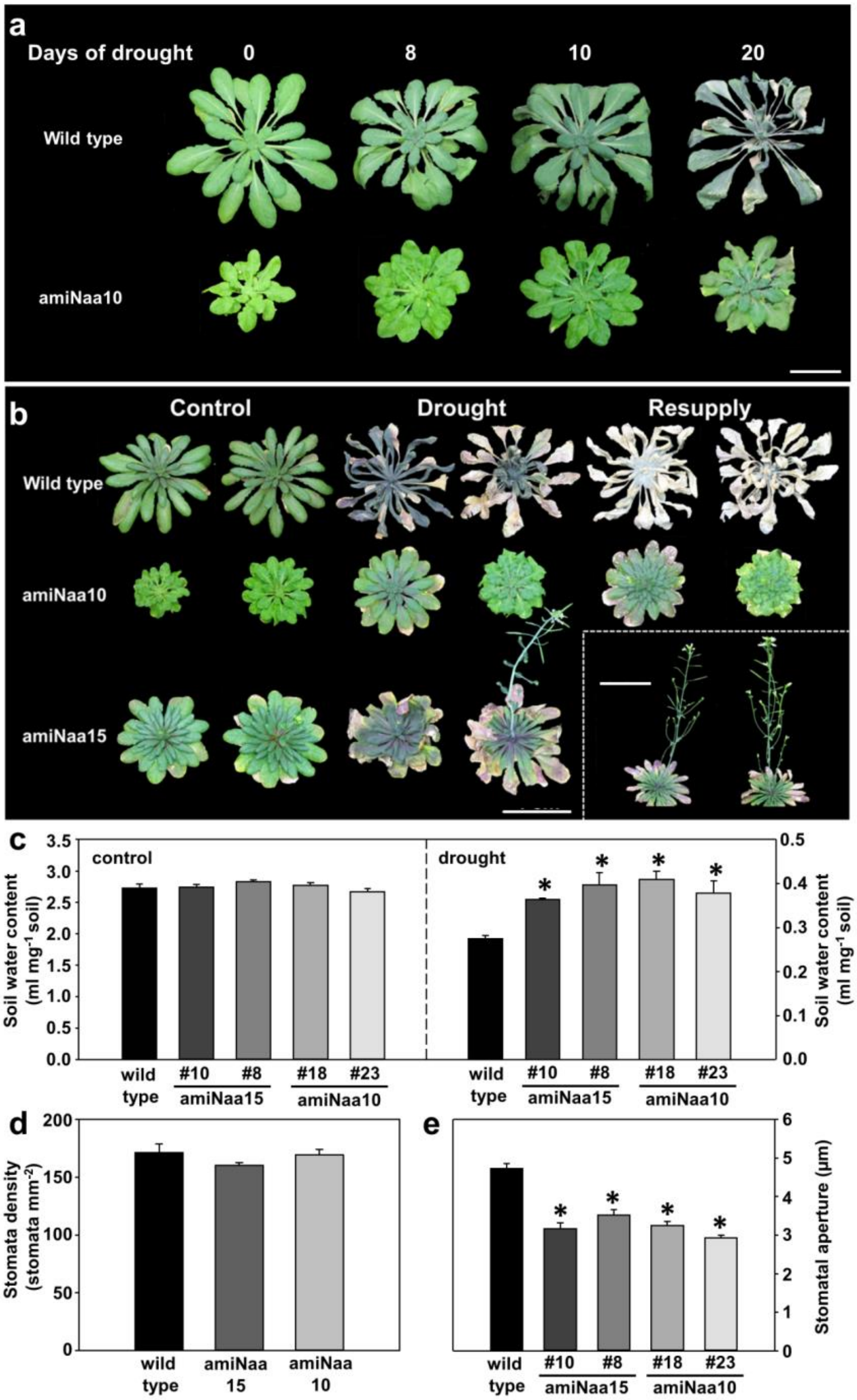

Supplementary Fig. 5: Growth phenotype and recovery of NatA depleted plants upon prolonged drought

a, b) Wild type and NatA depleted plants were grown on soil under short day conditions for six weeks and subsequently challenged with drought for indicated time. **a**) Prolonged drought caused typical wilt phenotype in the wild type in a time dependent manner. In contrast NatA depleted lines did not show any visible phenotypes. (Scale bar = 3 cm) **b**) After 20 days of drought the wild type died and could not be rescued with resupply of water. All analyzed NatA depleted lines almost stopped growth

under prolonged drought, but do not show visible symptoms associated with water deficiency. After resupply with water all NatA depleted plants continued to grow and produce viable seeds. (scale bars = 4 cm, 8 cm for right panel) **c**) Soil water content of wild type and NatA depleted plants grown on under short day conditions for six weeks. Plants were subsequently treated for 20 days with (left plot) or without water (right plot). Data are represented as mean ± standard error. Asterisks indicate significant differences to wild type ($p<0.05$, N = 4, Student's t-test). **d)** Density of stomata on leaves from wild type and NatA depleted grown on soil under short day condition with regular water supply for 6 weeks. Data are represented as mean ± standard error. Asterisks indicate significant differences to wild type. ($p<0.05$, N = 5, Student's t-test) **e)** Quantification of stomata aperture in wild type and NatA depleted plants. Data are represented as mean ± standard error. Asterisks indicate significant differences to wild type. ($p<0.05$, N = 10 - 17, Student's t-test)

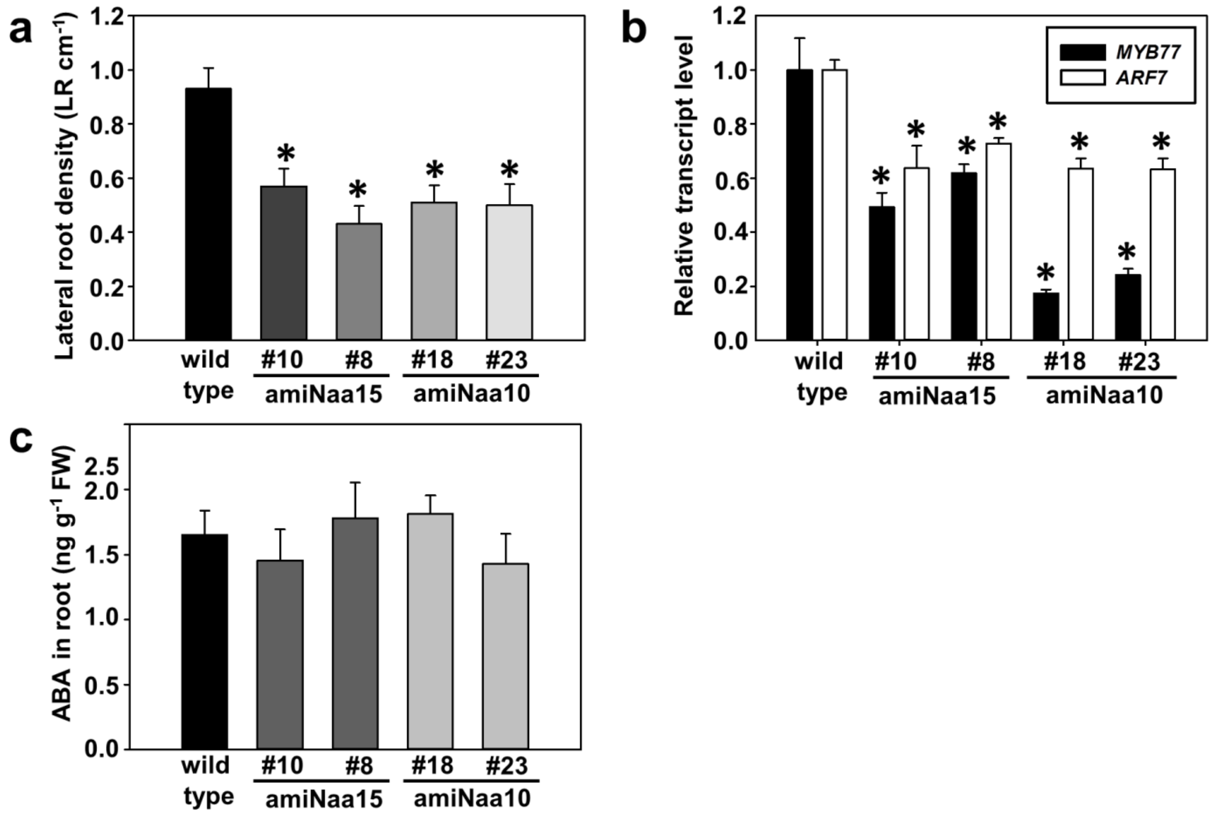

Supplementary Fig. 6: The NatA depleted plants display drought stress adapted root morphology

a) Quantification of lateral root density from 2-week-old wild type and NatA depleted plants grown under short day conditions on solid ½ MS-medium (shown in Fig. 4H). Asterisks indicate significant differences ($p<0.05$, N = 25 - 40). Data are represented as mean ± standard errors. **b-c)** Transcript steady state level of the lateral root formation controlling transcription factors, *MYB77* and *ARF7* (**b**), and ABA level (**c**) in roots of wild type and NatA depleted plants grown hydroponically on ½ Hoagland medium for 6 weeks under short day conditions. Asterisks indicate significant differences ($p<0.05$, N = 3-5). Data are represented as mean ± standard errors.

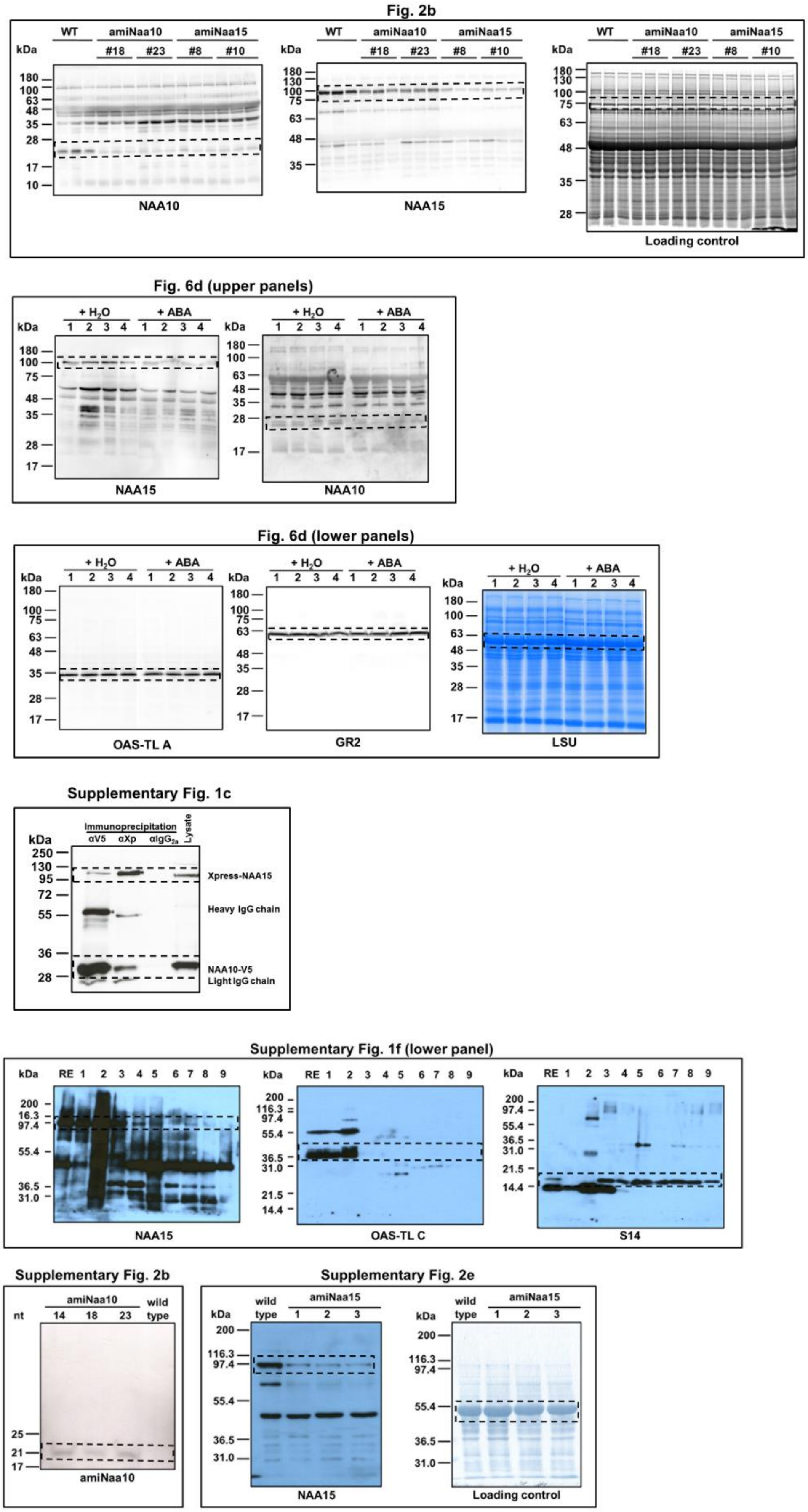

Supplementary Fig. 7: Original images of cropped figures shown in the paper

Supplementary Table 1: Primers used in this study

Name	Sequence (5'-3')	Purpose
NAA10P1	gatacatagccaacgatgcgacgtctctcttttgtattcc	amiRNAi genaration
NAA10P2	gacgtcgcatcgttggctatgtatcaaagagaatcaatga	amiRNAi genaration
NAA10P3	gacgccgcatcgttgcctatgtttcacaggtcgtgatatg	amiRNAi genaration
NAA10P4	gaaacataggcaacgatgcggcgtctacatatatattcct	amiRNAi genaration
NAA15P1	gatagtcatgcaatttcaggtggtctctcttttgtattcc	amiRNAi genaration
NAA15P2	gaccacctgaaattgcatgactatcaaagagaatcaatga	amiRNAi genaration
NAA15P3	gaccccctgaaattggatgactttcacaggtcgtgatatg	amiRNAi genaration
NAA15P4	gaaagtcatccaatttcagggggtctacatatatattcct	amiRNAi genaration
pRS300_A	ctgcaaggcgattaagttcggtaac	amiRNAi genaration
pRS300_B	gcggataacaatttcacacaggaaacag	amiRNAi genaration
NAA10_BamHI_f	gatcggatccatggtttgcatcaggcg	Antibody generation
NAA10_XhoI_r	gatcctcgagtcatttggaaactgctttacc	Antibody generation
NAA15_BamHI_f	gatctcgagcaaagaattaggtgaatgtttctggagt	Antibody generation
NAA15_XhoI_r	gatggatccggactctcccaagtcaactgct	Antibody generation
NAA10_B_f	atctcgagatggtttgcatcaggcgagc	BiFC
NAA10_B_r	atggtacctttggaaactgctttacc	BiFC
NAA15_B_f	atctcgagatgggggcttcgcttcctcc	BiFC
NAA15_B_r	atggtaccggttgcaactgagaggctcttg	BiFC
NAA10_C_f	ggggacaagtttgtacaaaaaagcaggctgcatggtttgcatcaggcgagcgacg	Complementation
NAA10_C_r	ggggaccactttgtacaagaaagctgggtctcatttggaaactgctttaccatctac	Complementation
AtNAA10_BsmBI_f	gatcatcgtctcccatggtgatggtttgcatcaggcg	Expression in *E. coli* cells
AtNAA15_BsmBI_r	gatcatcgtctcggtacctcatttggaaactgctttacc	Expression in *E. coli* cells
AtNAA10_f	agccatggtttgcatcaggcgag	Expression in mamalian cells
AtNAA10_r	tttggaaactgctttacc	Expression in mamalian cells
AtNAA15_f	atgggggcttcgcttcc	Expression in mamalian cells
AtNAA15_stop_r	tcaggttgcaactgagag	Expression in mamalian cells
NAA10_LP	ctgccatggaacaggttactc	Genotyping
NAA10_RP	gggaaaactgtacaagccaag	Genotyping
NAA15_1_LP	gtttctctcaacacagagccg	Genotyping
NAA15_1_RP	accactggcagttgattcttc	Genotyping
NAA15_2_LP	gtggatgcctatgttggtacg	Genotyping
NAA15-2_RP	tgccaaagataatgaagcagg	Genotyping
NAA20_1_LP	ggctggctctttttgattttc	Genotyping
NAA20_1_RP	ttctacatggtttcagttatggg	Genotyping
SAIL_BP	tagcatctgaatttcataaccaatctcgatacac	Genotyping
SALK_BP	gaccgcttgctgcaactctctcagg	Genotyping
WISC_BP	aacgtccgcaatgtgttattaagttgtc	Genotyping
NAA10_L_f	ggggacaagtttgtacaaaaaagcaggctgcatggtttgcatcaggcgagcgacg	Localization
NAA10_L_r	ggggaccactttgtacaagaaagctgggtctttggaaactgctttaccatctac	Localization
NAA15_L_f	ggggacaagtttgtacaaaaaagcaggctgcatgggggcttcgcttcctcctaa	Localization
NAA15_L_r	ggggaccactttgtacaagaaagctgggtcggttgcaactgagaggctcttgaa	Localization
Actin7_f	caaccggtattgtgctcgattc	qRT-PCR

Actin7_r	aacctcaggacaacggaatctc	qRT-PCR
amiNaa10_f	tacatagccaacgatgcgac	qRT-PCR
ARF7_f	cgaagggtctttcatctctaag	qRT-PCR
ARF7_r	ctttctacatatgtcctcctcc	qRT-PCR
LEA7_f	caagaacagagttacaaagctgg	qRT-PCR
LEA7_r	actgggctgtctcttgagtttt	qRT-PCR
MYB77_f	cgaactcgtgttctcagctc	qRT-PCR
MYB77_r	gtggttccgcagccgttac	qRT-PCR
NAA10_f	cggtctcgccactaagctc	qRT-PCR
NAA10_r	ctcggttacttctcctcacat	qRT-PCR
NAA15_f	gttggtcgtctagaagaagcca	qRT-PCR
NAA15_r	tagcttcatcggcaagtgcag	qRT-PCR
PP2A_PDF2_f	cttctcgctccagtaatgggatcc	qRT-PCR
PP2A_PDF2_r	gcttggtcgactatcggaatgagag	qRT-PCR
TIP41_like_f	gatgaggcaccaactgttcttcgtg	qRT-PCR
TIP41_like_r	ctgactgatggagctcgggtcg	qRT-PCR
NAA10_Y_f	atcatatggtttgcatcaggcgagc	Yeast two hybrid
NAA10_Y_r	atggatcctcatttggaaactgctttac	Yeast two hybrid
NAA15_Y_f	atgaattcatgggggcttcgcttcctcc	Yeast two hybrid
NAA15_Y_r	atggatccttacgtatgagcaatggcctcatc	Yeast two hybrid

Supplementary references

1. Marty, L. *et al.* The NADPH-dependent thioredoxin system constitutes a functional backup for cytosolic glutathione reductase in Arabidopsis. *Proc. Natl. Acad. Sci. USA* **106**, 9109-9114 (2009).
2. Arnesen, T. *et al.* Proteomics analyses reveal the evolutionary conservation and divergence of N-terminal acetyltransferases from yeast and humans. *Proc. Nat. Acad. Sci.USA* **106**, 8157-8162 (2009).
3. Goetze, S. *et al.* Identification and functional characterization of N-terminally acetylated proteins in Drosophila melanogaster. *PLoS Biol.* **7,** e1000236 (2009).
